# Transgenic hepatitis B: a new model of HBV infection

**DOI:** 10.1038/s41598-017-02862-2

**Published:** 2017-06-01

**Authors:** Hubert D.-J. Daniel, Michael Torbenson

**Affiliations:** 1 Johns Hopkins University School of Medicine, Department of Pathology, 733 N Broadway, Baltimore, MD 21205 USA; 2 Mayo Clinic, Hilton Building, 11th floor, 200 First Street SW, Rochester, MN 55905 USA

## Abstract

Chronic hepatitis B infection (HBV) is major cause of morbidity and mortality throughout the world. Currently there is limited understanding on the cellular proteins and related molecules involved in the critical steps of viral entry into the cytoplasm and persistent viral replication in cell culture. In order to address these fundamental questions, we designed and implemented a new model of hepatitis B: infectious transgenic hepatitis B virus composed of a complete virus plus a foreign gene. The foreign gene allows identification of cells that are infected by the transgenic virus. The transgenic virus was used in a functional assay to identify cellular proteins necessary for viral replication. This assay repeatedly identified the protein UQCR10. After restoring UQCR10 levels in HepG2 and Huh7 cells, they can be infected by intact virions of transgenic hepatitis B. These results demonstrate the usefulness of this new transgenic hepatitis B model.

## Introduction

Hepatitis B virus (HBV) infection is a global public health problem with over 350 million individuals infected worldwide. In the United States, 1.25 to 2 million individuals have chronic HBV^1^. The substantially morbidity and mortality caused by chronic HBV are well known in the medical and scientific communities and include the development of liver cirrhosis, liver decompensation, and liver cancer. Current treatments are limited and provide few individuals with sustained, long term reduction in viral replication. Even fewer individuals achieve sustained viral clearance. Thus, new treatments are needed for chronic HBV. New treatment approaches are most likely to succeed when they are firmly grounded in scientific understandings of viral biology. In this regard, much of the details on the biology of HBV remains unknown. While seminal studies have now identified the HBV receptor as sodium taurocholate co-transporting polypeptide^2^, encoded by *SLC10A1*, data is sparse on the other cellular proteins and related molecules that are critical to viral entry and sustained viral replication.

In order to study these key issues, we developed a new transgenic model of hepatitis B. This tool allowed the use of a functional approach to identifying key viral entry factors/early replication factors, an approach that was solely based on viral entry and replication in cells and was not dependent on *a prior* knowledge of necessary viral entry or post-entry factors necessary for sustained infectivity.

## Results

### Transgenic HBV: A new model of HBV infection

A new model transgenic model of hepatitis B was developed, where the hepatitis B virus expresses foreign proteins. Transgenic hepatitis B viruses were created with antibiotic resistance genes or green fluorescent protein genes. The foreign gene contained by the transgenic hepatitis B virus allows selection for subgroups of cells that have become infected by the transgenic virus.

**Figure 1:**
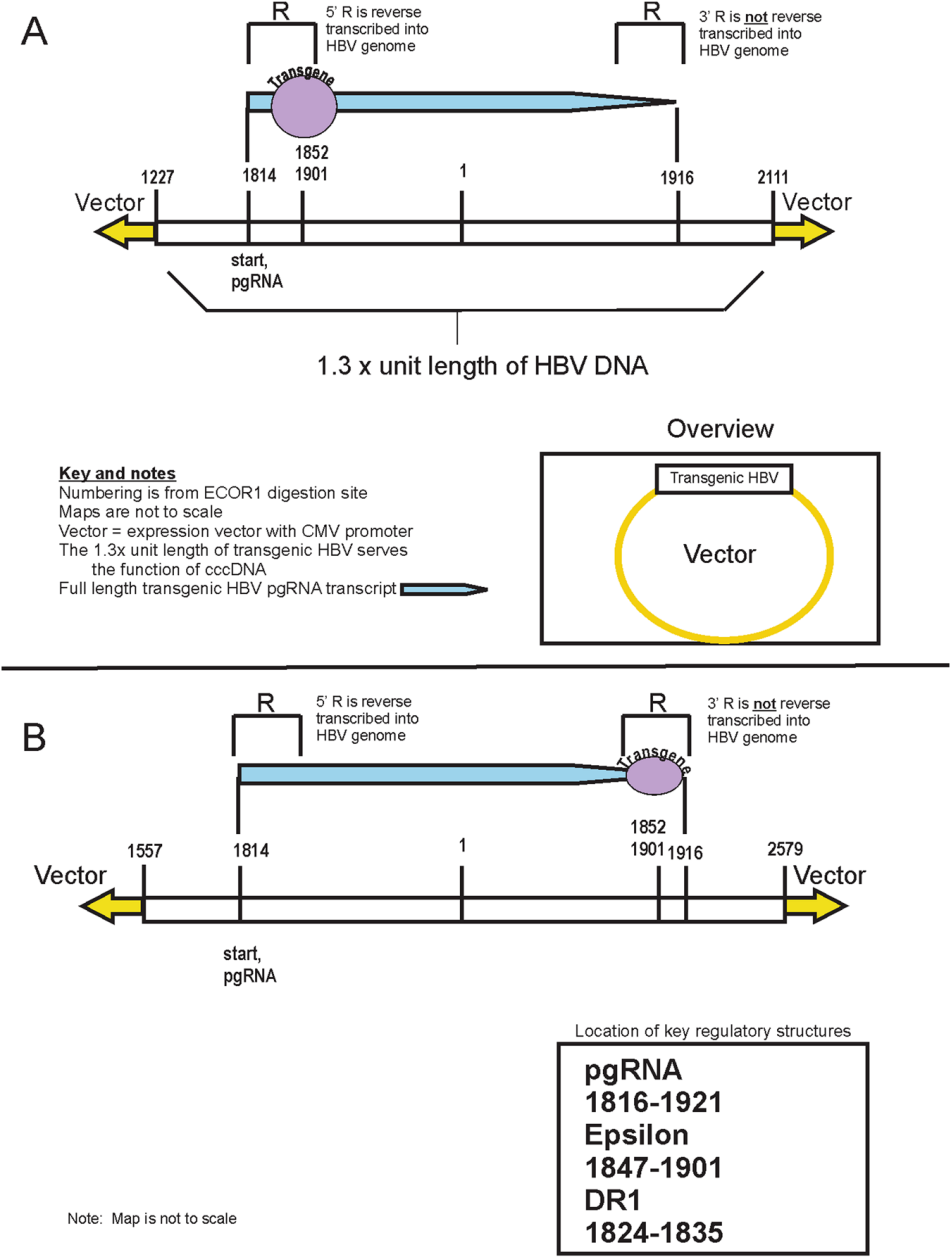
Transgenic HBV maps. Panel (A) Map of the “front” transgenic HBV, where the foreign gene is inserted within the first R region. Sequence numbering is from the traditional EcoR1 digestion site in the hepatitis B genome. Panel (B). Map of the “back” transgenic HBV, where the foreign gene is inserted within the first R region.

Insertion of a foreign gene into the HBV genome without disrupting viral gene expression is particularly challenging because the HBV genome has overlapping open reading frames. Thus, insertion at a given location may avoid disrupting one gene, but will disrupt other genes coded in overlapping reading frames. However, based on analysis of the HBV genome and empirical observations, we successfully inserted foreign genes at nucleotide sites 1852 base pair (bp) or 1901 bp (Fig. 1). Consideration of the known HBV genetic map reveals the following: (1) insertion of new genes at 1852 bp or 1901 bp locations does not interrupt the P, S, X, or C open reading frames; (2) DR1 and DR2 are not affected and the epsilon region is not affected (1901 insertion site) or affected only at the outer edge of the Epsilon coding region (1852 insertion site). Epsilon is a segment of the pgRNA that forms a hairpin secondary structure that is necessary for pregenomic RNA (pgRNA) encapsidation and for DNA replication; (3) pgRNA is affected and increases in length because of the transgene; (4) the HBeAg open reading frame is affected by both insertion sites, although the affected part is normally cleaved off in the Golgi prior to secretion of HBeAg for the 1852 insertion site^3^. Vectors with full 1.3× length transgenic hepatitis B genomes, including both insertion sites, secrete HBeAg (Supplemental Table 1).

In order to create packaged, infectious, transgenic virions, we constructed greater-than full length transgenic viruses (1.3× full length) and cloned them into a mammalian expression vector (Materials and Methods). This 1.3× unit length of transgenic HBV DNA serves the function of covalently close circular DNA (cccDNA). cccDNA is the form of viral DNA that allows production of infectious virions by serving as the template for the production of pgRNA. pgRNA is a greater-than-full-length transcription of cccDNA that is packaged into virions and subsequently reverse transcribed into viral DNA. Because the pgRNA is created by transcribing the entire circular loop of cccDNA, plus an extension beyond the initial start point, pgRNA contains two terminal redundancies, denoted as “R,” which are located at both the 5′ and 3′ end of the pgRNA. The R contains key viral DNA regulatory sequences including DR1 as well as the epsilon region. In normal HBV replication, the 5′ R will be incorporated into the viral cccDNA while most of the 3′ R will not. Of note, the transgene insertions sites of 1852 and 1901 are both in the R region. Thus, there are two ways to construct the 1.3× transgenic viral genomes. In the first construct, the transgene is inserted in the 5′R (Fig. 1A), while in the second, the transgenes is inserted in the 3′R (Fig. 1B). In the second model, the transgene is included in the pgRNA but not in the fully reverse transcribed viral DNA. These designs have different outcomes: the first construct produces fully packaged transgenic HBV, while the second construct packages wild type virus. The first transgenic HBV design was used in subsequent assays for viral entry factors.

### Transgenic HBV as a functional assay identifies UQCR10

**Figure 2:**
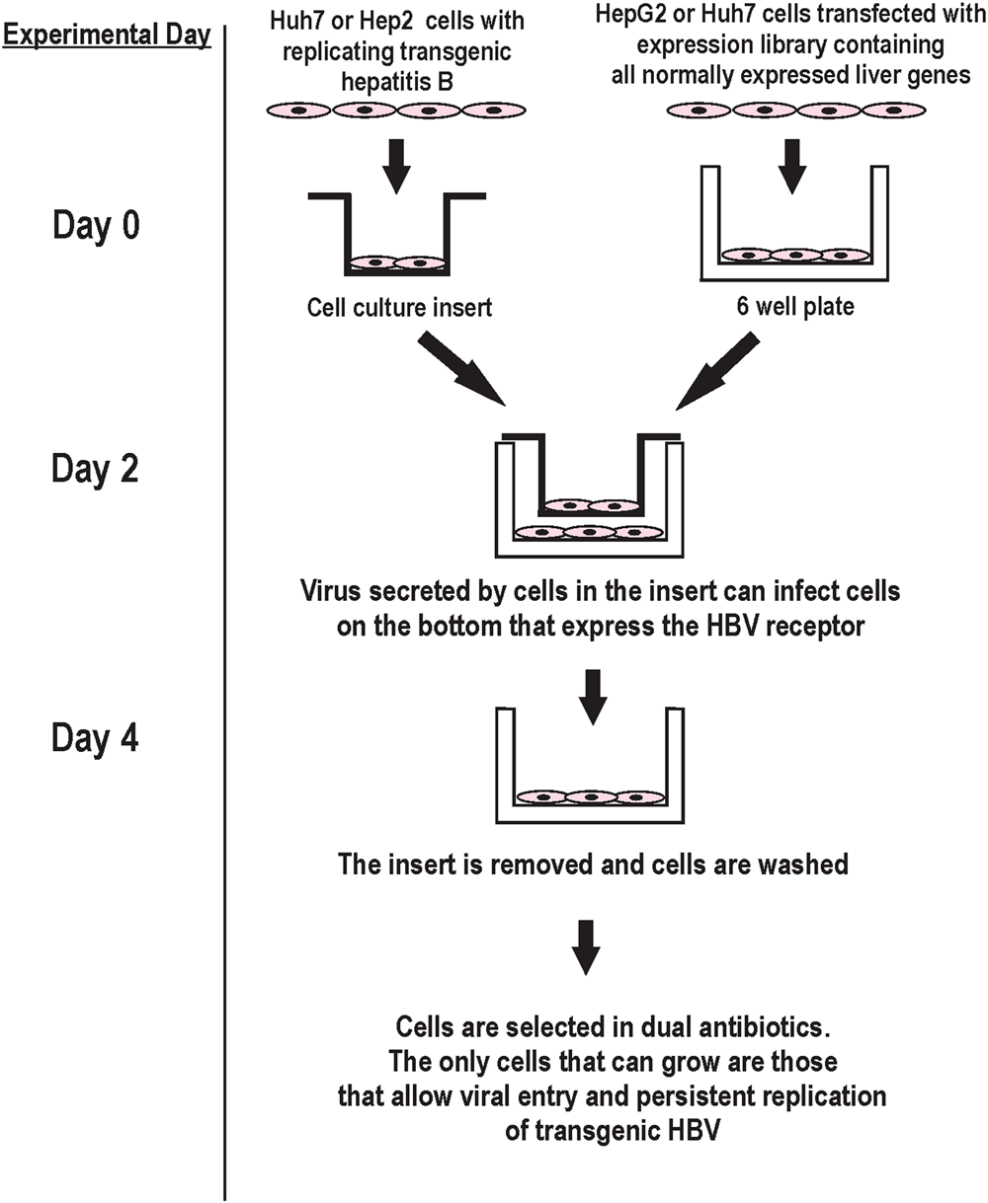
Experimental outline. Assay used to identify viral entry/replication factors.

We designed a functional assay to identify unknown viral entry/persistent replication factors (Fig. 2 and Supplemental Figure 1). In this assay, this unknown protein(s) is restored to the liver cell lines HepG2 or Huh7 by transfection of a gene expression library that was made from mRNA extracted from histologically normal human liver tissue. After this transfection, a random subset of the HepG2 or Huh7 cells are anticipated to express the missing factor(s) needed for viral entry/early replication. In the next step of the assay, these library-transfected cells are exposed to infectious transgenic virions for 3 days. The cell lines in the cell culture well inserts (Fig. 2) secrete transgenic virus into the media, which is shared by the cells that were transfected with the liver expression library. Physical barriers keep the cells completely separated. The long exposure was chosen because published data suggests relatively slow viral entry. After this, antibiotics are added to select for those cells that have become infected with transgenic hepatitis B virions. Most cells die (>99%), but cells will survive if they express factors from the expression library that permitted entry or sustained replication of the transgenic virus encoding the antibiotic resistance gene. For example, blasticidin added to the cell media will kill cells if they do not (1) express proteins that allow entry of the transgenic virus, (2) have functional transgenic virus that produces and secretes the antibiotic resistance gene. After the cells are fully selected in antibiotics, cellular DNA is harvested and the library vector identified by PCR and sequencing.

To estimate the percent of cells that would pick up transgenic virus in this assay, a shortened version of the full assay was first performed in Huh7 cells in duplicate with transgenic virus encoding a green fluorescent protein. Flow cytometry at 72 hours, demonstrated a small percentage of library transfected cells (0.6 ± 0.2%) that were strongly GFP positive. This data encouraged us by providing evidence for the infectivity of the transgenic virus and provided an estimate of the percent of cells that express either the receptor or factors necessary for sustained replication.

The assay (Fig. 2) was then repeated on 4 separate occasions using two different transgenic viruses encoding two different antibiotic resistance genes and both HepG2 and Huh7 cell lines. In each experiment, the fully selected cells at the end of the assay contained a library vector with DNA encoding the UQCR10 gene (Supplemental Tables 2 and 3). The fully selected cells were also positive for HBsAg (Supplemental Figure 2). Examination of the original liver gene expression library by real time PCR showed that UQCR10 made up 0.5% of the original library.

Control conditions ruled out nonspecific uptake of transgenic viral DNA by the library-expressing cells (Supplemental Table 2). To rule out UQCR10 selection that was independent of viral infection, additional experiments were performed in which the library alone was transfected into wild type cell lines, followed by long term selection in Geneticin (the library vector encodes the antibiotic resistance gene for Geneticin): no enrichment for UQCR10 was seen.

### Confirmation of UQCR10 as a viral replication factor

**Figure 3:**
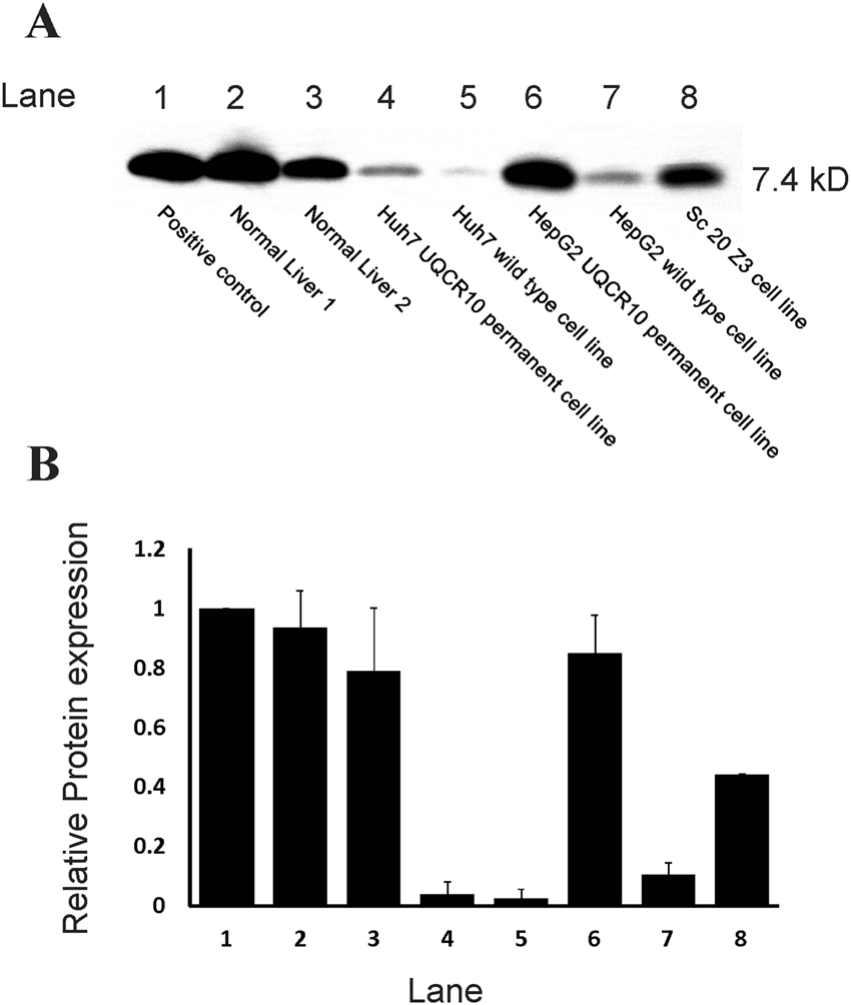
Panel (A) Western blot for UQCR10 protein. Lane 1: Positive control consisting of mitochondrial protein extracted from human heart (MitoSciences, Eugene, Oregon, USA); Lane 2: representative normal liver; Lane 3; second case of representative normal liver; Lane 4: Huh7 cell line permanently expressing UQCR10; Lane 5, wild type Huh7; Lane 6: HepG2 cell line permanently expressing UQCR10; Lane 6, wild type HepG2; Lane 7, HepG2 cell line selected out by assay experiment. Panel (B) Densitometry of lanes in Panel 3 (A) Protein levels were normalized to the control in lane 1. Two independent blots were analyzed. The average ratio of the test/control (±standard deviation) is shown.

**Figure 4:**
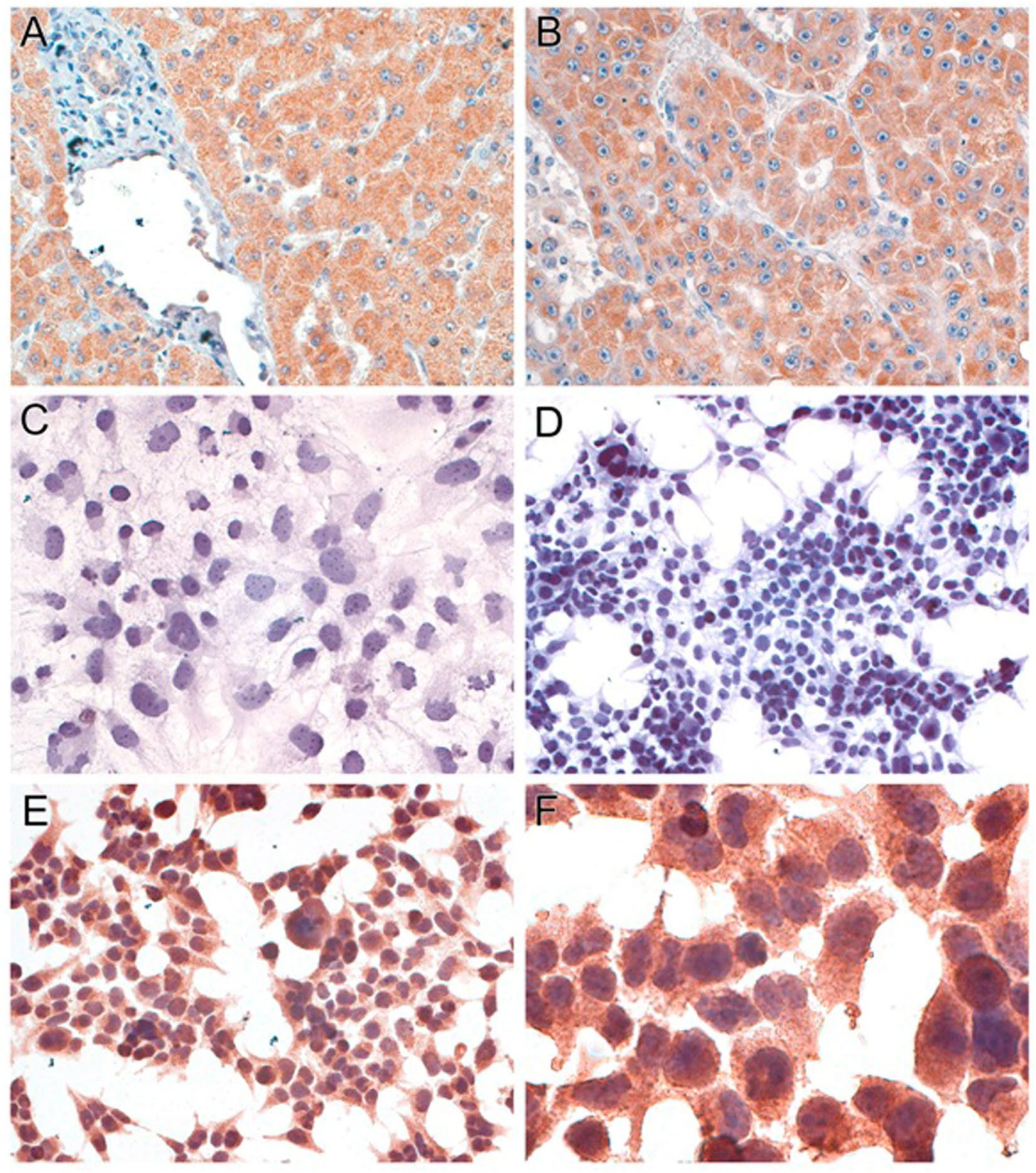
Immunohistochemistry for UQCR10 expression. Panel (A) (original magnification, 160×), Non-neoplastic hepatocytes express UQCR10; Panel (B) (160×), Hepatocellular carcinoma expresses UQCR10; Panel (C) (160×), Wild type Huh7 cell lines have little or no UQCR10 expression; Panel (D) (100×), Wild type HepG2 cell lines have little or no UQCR10 expression; Panel (E) (160×), HepG2 cells obtained at the end of the assay express UQCR10; Panel (F) (400×), Higher magnification from same cells in panel E showing granular cytoplasmic staining.

To directly determine a role for UQCR10 in HBV infection, we analyzed UQCR10 levels in human tissues and HepG2 and Huh7 cell lines and tested whether restoration of UQCR10 protein levels in these cell lines would make them susceptible to sustained infection. First, UQCR10 mRNA was measured in HepG2 and Huh7 cell lines: surprisingly, the levels were similar to normal liver tissues (Supplemental Table 4). However, protein levels were significantly lower in HepG2 and Huh7 cell lines than in human liver tissues (Fig. 3), suggesting a post transcriptional inhibitor of UQCR10 expression. Protein levels were not restored by temporary transfection but were partially restored by creating permanent cell lines containing UQCR10 cloned into an expression vector (Fig. 3, lanes 4 and 6). In addition, one of the cell lines that grew from one of the assay replicates (laboratory designation sc20 Z3) was also tested and it expressed high levels of UQCR10 protein (Fig. 3, lane 8). Immuno-histochemistry confirmed these findings: UQCR10 protein expression was seen in both the cytoplasm of non-neoplastic liver tissues (Fig. 4A) and in the cytoplasm of hepatocellular carcinoma (Fig. 4B), but showed low to absent expression in HepG2 and Huh7 cell lines (Fig. 4, panels C,D). UQCR10 protein expression levels were also positive in cell line sc20 Z3 (Fig. 4, panels E,F).

**Figure 5:**
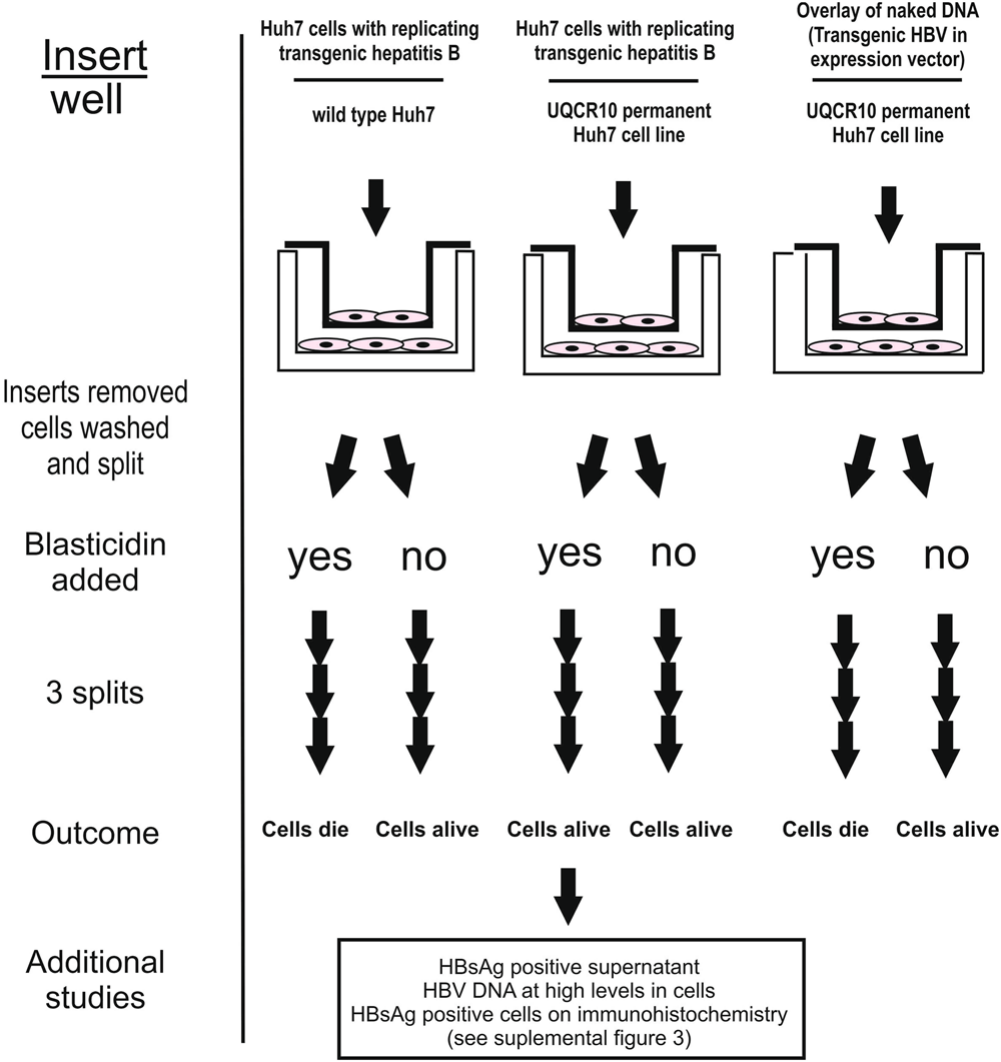
Transgenic confirmation studies. Experimental outline and results, which demonstrates that permanent UQCR10 Huh7 cell lines are permissive for sustained infection by transgenic HBV.

We next investigated whether HepG2 and Huh7 cell lines with permanent expression of UQCR10 protein could be infected by transgenic hepatitis B particles. After exposing Huh7 cells to transgenic virions, transgenic virus was able to enter and replicate in cells that express UQCR10 but not in wild type cell lines (Fig. 5). HBsAg was also detectable by ELISA in the cell culture supernatant and in the cell cytoplasm by immunohistochemistry, confirming viral entry and subsequent protein production (Supplemental Figure 3, panel A).

Control wells were exposed to plasmid constructs with the 1.3× transgenic viral DNA instead of the secreted transgenic virions and all of these died in the presence of antibiotic, demonstrating that entry of transgenic viral DNA into the permanent UQCR10 cell lines required the association of viral proteins.

Without the presence of antibiotic pressure (Fig. 5), viral protein expression levels were low in the UQCR10 cell lines (Supplemental Figure 3). This observation suggests that viral infection does not necessarily lead to long term viral retention in rapidly dividing cell lines without the presence of selective pressure to retain the virus. Also of note, the control wild type Huh7 cell lines from the experiment (Fig. 5) were clearly HBsAg and HBV DNA positive at the end of the experiment (Supplemental Figure 3, panel D and Supplemental Table 5), indicating low level viral entry in wild type cell lines, as has been observed by others^2^. However, without UQCR10, the HBV DNA levels consistently fell, split-over-split until they were nearly absent by the experimental end (Supplemental Table 5).

We next tested whether transgenic viral infection and sustained replication was supported by UQCR10. In this experiment (Supplemental Figure 4), a permanent UQCR10 HepG2 cell line was used, but instead of a wild type control, we used a second UQCR10 cell line that was previously created but showed low expression of UQCR10 by western blot. Most permanent cell lines have heterogeneity at the individual cell level for expression of the vector encoded gene, in this case UQCR10, and we reasoned that this could be exploited to study the role of UQCR10. If UQCR10 plays an important role in sustaining persistent viral replication, we hypothesized that transgenic virus should enter many different cells within the low-UQCR10 producing cell line, but should replicate better in those cells with higher levels of UQCR10 expression. Thus, HBV sustained infection should preferentially occur in the subpopulation of cells within the low-UQCR10 producing cell line that have relatively higher UQCR10 expression. It then follows that daughter cells selected at the end of the experiment should produce more produce more UQCR10. The experimental results were as predicted; indicating UQCR10 enhanced viral replication (Supplemental Figure 4).

Prior research has firmly established a role for the viral preS1 protein in binding the cell receptor. Given this, we studied whether the entry of transgenic virus into UQCR10 positive cell lines was also dependent on the viral pre-S1 protein. Blocking studies with pre-S1 antibodies strongly inhibited transgenic infection of cell lines (Supplemental Figure 5)

Lastly, to confirm a role for UQCR10 with infection from natural hepatitis B virions, UQCR10 permanent cell lines were exposed to unmodified human serum from individuals infected with chronic hepatitis B. Low but persistent viral protein production was observed for as long as 12 splits (Supplemental Figure 6). However, viral DNA, as measured by real time PCR, was barely detectable in both cell culture supernatants and cellular extracts. The lack of infection with robust DNA levels supports the hypothesis that additional pressure is required for cells to retain the virus. Additional studies will be needed to more fully define the role of UQCR10.

### Functional studies of UQCR10

We next studied whether UQCR10 expression may stimulate downstream targets that do have a role in physical entry of the virus or in early replication. To explore this further, genome wide expression studies were performed to compare the gene expression patterns of UQCR10 permanent HepG2 and Huh7 cell lines to their respective wild type controls. Analysis of changes in individual genes and in expression pathways did not identify the know HBV receptor sodium taurocholate co-transporting polypeptide, suggesting UQCR10 plays a role in sustained viral replication after viral entry into the cell (Supplemental Table 6).

Prior studies have suggested that “maturation” of cell lines increases HBV replication. To investigate if UQCR10 affected cellular maturation, mRNA levels of 6 different genes associated with a mature hepatocyte phenotype were measured. Interestingly, UQCR10 had opposite effects on the two studied cell lines, leading to increased expression of genes associated with a mature hepatocyte phenotype in Huh7 but not in HepG2 (Supplemental Table 7). UQCR10 expression also did not affect ATP production, apoptosis, phagocytic activity, or proliferation (Supplemental Table 8). However, UQCR10 expression clearly enhanced HBsAg secretion into the supernatant after temporary transfection in HepG2 cells (Supplemental Table 8). Together, these data suggest that UQCR10’s role in HBV infection does not primarily involve non-specific functions such as increasing the maturation of the cell lines, increasing energy production, increasing phagocytic activity, or changing the balance of apoptosis and proliferation in these cell lines. Instead, UQCR10 expression increases the level of viral protein production/secretion.

### Additional unique aspects of the transgenic HBV model

Analysis of miniprep DNA from fully selected cells in the Sc16 assay (Supplemental methods) revealed an additional surprise. Sequencing of clones obtained from electroporation of bacteria showed that most were positive for UQCR10. However, a subset of the clones contained unexpected configurations of the transgenic HBV and its vector. Sequencing of the DNA indicated that in some instances pgRNA had started at the second pgRNA start site in the 1.3× transgenic DNA construct and extended around the full length of the vector to end at the first stop sequence in the HBV genome (Supplemental Figure 7, transcript No. 3). This DNA molecule was designated “partial virus-full vector” and could be reproduced by temporary transfection of the full 1.3× vector-plasmid construct into HepG2 cells, followed by selection in geneticin, for which the vector has an antibiotic resistance gene. Geneticin provides pressure to retain vector DNA and sequencing of plasmids obtained by miniprep under these conditions showed the exact partial virus-full vector sequence predicted by use of this alternative start and end site of pgRNA. These viral-vector configurations would be unable to self-replicate through the normal HBV pathways, due to the absence any complete viral proteins, and are thus “dead-end” or “defective” viruses.

Further analysis of the fully selected cell lines from the Sc16 assay (Supplemental Table 2) was also surprising because the fully selected cells had high levels of both transgenic viral DNA as well as the plasmid DNA in which the transgenic virus had been cloned (pcDNA3.1). This finding raised the possibility that the assay result represented nonspecific uptake of the plasmid released from dying cells or plasmid that had not been fully washed away prior to putting the insert into the wells (Fig. 2). However, repeat experiments showed the same result, and cell lines selected by the assay were positive for UQCR10 as well as high levels of HBV DNA and pcDNA3.1 plasmid DNA. The repeated finding of UQCR10 in multiple assays and in two different cell lines is inconsistent with random or nonspecific uptake of the transgenic virus-plasmid construct. Furthermore, additional controls with high levels of naked plasmid-1.3× transgenic viral DNA overlaid on the cells with the liver expression library failed to grow out cells (Supplemental Table 2), ruling out nonspecific uptake of naked DNA. Based on our experience with alternative viral transcripts leading to “partial virus-full vector” molecules, we hypothesized that this reflects full packaging of the 1.3× virus and the full vector (Supplemental Figure 7, transcript 2). These novel transcripts are best explained by the transgene having a repressive effect on the 5′ epsilon in the transgenic HBV-plasmid construct, leading to preferential usage of the second pgRNA start site and the second unmodified epsilon for viral nucleic acid packaging (Supplemental Figure 7, transcripts 2,3). Prior studies have shown that disruption of the 5′ epsilon substantially impairs pgRNA encapsidation^4–6^. Regardless of the mechanism, taken together these results indicate an unanticipated ability of HBV replication machinery to reverse transcribe large segments of foreign nucleic acid that have been packaged into viral capsids as part of transgenic pgRNA molecules.

We also sought to create cell lines that would more closely mimic wild type infection by containing non-integrated viral cccDNA as the template for producing virions. These were created by amplify full length virus using previously described methods^7^. Transfection of these full length transgenic viral DNAs into HepG2 and Huh7, followed by selection with the transgenic antibiotic, created cell lines with transgenic viral cccDNA but no plasmid (Supplemental Table 9). Also of note, high levels of antibiotic could be used to cause rapid viral integration and within 4 splits little non-integrated virus was detected by miniprep of cell extracts.

## Discussion

Transgenic hepatitis B represents a new model of hepatitis B infection. The usefulness of this model is demonstrated by the identification of UQCR10 as a key factor for viral replication. The transgenic HBV model also explains the lack of persistent infection when HepG2 and Huh7 cell lines are exposed to wild type virus. Viral infection is not persistent because (1) viral protein expression is insufficient in the setting of diminished UQCR10 protein expression and (2) viral DNA is quickly lost in the rapidly dividing cell lines. Transgenic hepatitis B models overcome this latter limitation by allowing antibiotic selection pressure that forces cell lines to retain the virus.

As an unanticipated result, this model has further illustrated the ability of HBV to package relatively large nucleic sequences using the second epsilon as a start site. In addition, modifications of this model can uniquely produce virions in the same manner as natural HBV infections—using viral cccDNA as the template, without the need for plasmids. We anticipate this model will be useful in dissecting the molecular biology of viral replication and in screening for new drugs. This model is further enhanced by the substantial flexibility where a wide variety of genes can be inserted into the viral genome and, through infection, into hepatocytes, potentially providing a novel platform for gene delivery.

We demonstrated the utility of the transgenic hepatitis B by using it as a functional assay to identify viral replication factors. These studies identified UQCR10 as an important viral replication factor. UQCR10 is a subunit of mitochondrial complex III, also called the ubiquinol-cytochrome c reductase complex. Mitochondrial complex III forms the middle segment of the respiratory chain of the inner mitochondrial membrane^8^. These results fit well with other published observations. First, reduced UQCR10 levels in the hepatocellular carcinoma cell lines HepG2 and Huh7 is in keeping with the observation that complex III proteins are reduced in human hepatocellular carcinomas^9^. Secondly, glucocorticoids are known to increase HBV replication in HepG2 cells^10^ and glucocorticoids increase mitochondrial complex III activity in HepG2 cells^11^. Thirdly, ddC reduces all mitochondrial proteins, including complex III^12^, and inhibits HBV replication in a HepG2 model^13^.

In addition, other studies have demonstrated that mitochondrial complex III binds to the hepatitis B X protein^14, 15^, with recent studies mapping the precise interaction site to amino acids 72–117 of HBx^16^. It is also interesting to note that some herbal remedies used to treat hepatitis B appear to work through interaction with the ubiquinol-cytochrome c reductase binding protein^17^. Finally, HBx gene knock in mice show increased expression of complex III in hepatocellular carcinomas^18^.

In sum, transgenic hepatitis B represents a new and powerful model to study viral replication and to identify key host factors. The use of this model in a functional based assay identified UQCR10 as host protein critical for viral replication. The flexibility of this model holds promise as a tool with far reaching applicability, similar to transgenic animal models that have been successful tools for understanding a wide range of cellular processes in normal and tumor biology.

## Methods

All experiments were performed at Biosafety Level 2

### cDNA synthesis

Total RNA was extracted from cells using TRIzol (Invitrogen life technologies, Carlsbad, CA, USA) followed by precipitation with isopropyl alcohol as per manufacture’s protocol. Two μg of RNA was used and cDNA was synthesized with oligo-dT primers using the Superscript First –Strand synthesis system for RT-PCR (Invitrogen) according to the manufacturer’s instructions in a 20 μl reaction. Two μl of cDNA was then used as input for real time PCR. Primers were designed using the Genebank gene data and were designed to cross exons. None of the primer sets amplified genomic DNA.

#### 5′ and 3′ RACE

RNA was extracted using TRIzol (Invitrogen) and was further purified using RNAeasy (Qiagen, Valencia, CA, USA). RACE was performed as per the manufacturer’s instructions (GeneRACER,). After cloning, samples were sequenced and aligned using BioEdit.

### Real time PCR

Real time PCR was performed with the SmartCycler system (Cepheid, Sunnyvale, California, USA) using the Fast Start SYBR green master mix (Roche, Indianapolis, IN) with 2 μl of cDNA or μl of DNA and cycling conditions of 95 °C for 10 minutes followed by 35 cycles of 95 °C for 20 seconds, 55 °C 30 seconds, and 72 °C for 30 seconds. The specificity of PCR products were ascertained by melt curve analysis. Expression levels were normalized to beta-glucuronidase for gene expression. To quantify HBV DNA levels, an absolute standard curve was used.

### Affymetrix

Western blots were performed on cellular extracts to confirm the over-expression of UCQR10 in each UQCR10 cell line compared to its wild type mate. These cell lines were then used for gene expression analysis. RNA was extracted using TRIzol (Invitrogen life technologies, Carlsbad, CA, USA) and was further purified using RNAeasy (Qiagen, Valencia, CA, USA). Gene expression was analyzed using the Illumina HT12 Expression array and was performed at the Johns Hopkins Sidney Kimmel Cancer CORE Facility. The core facility performed all of the labeling, hybridization, and scanning and provided analysis assistance. The wild type cell lines were compared to the same cell line with permanent UCQR10 expression.

For Affymetrix, each cell line was tested in duplicate. The PCA score and heat maps were used to assess data quality. The replicates where also checked using scatter plots to ensure similar transcript levels. The non-normalized sample data was used to generate box plots of the log AVG signal to ensure even distribution of the data. Ambiguous florescent signal data was removed and the remaining data was averaged and used to analyze the expression levels in both UQCR10 positive cell lines and in wild type cell lines. The data was analyzed using Genespring. Gene over-expression was defined using a signal log ratio 2, which correlates to a 4-fold increase in expression. Gene under-expression was defined using a signal log ratio −2.

### Transfection

Huh7 or HepG2 cells (American Type Culture Collection) were seeded at a density of 5.5 log cells in standard 6 well plates and grown overnight in Dulbecco’s modified Eagle’s Medium (DMEM with high glucose) with 10% fetal bovine serum (FBS) and transfected (Lipofectamine, Invitrogen, Carlsbad, CA) with 2 μg of DNA. The cells were washed and the growth medium replaced at 24 hours after transfection.

### Miniprep on cells

Plasmid DNA was isolated from cells using the Qiagen Mini-prep kit with modifications. After the addition of N3 neutralization buffer, the sample is incubated on ice for 5 minutes and then centrifuged at 13000 rpm for 10 minutes. The sample is then incubated for 5 minutes at 37 °C and centrifuged for 1 minute. Plasmid DNA is Eluted in 100uL of TE buffer.

### Cell proliferation Assay using WST-1 (Roche Applied Sciences)

HepG2 and Huh7 cell lines were grown in tissue culture plates in MEM with 10% FBS. The cells were trypsinized after reaching 80% confluence and 5 × 10^4^ cells were plated in wells of 96 micro titer plates. The plates were incubated at 37 °C for 24 hour and the reagent used as per the manufacturer’s instructions. The percentage of dye uptake, a measure of the number of viable cells, was determined spectrophotometrically at 450 nm and 600 nm wavelength at 0.5, 1, and 2 hours.

### Western blot

Total protein was extracted from cells as per the manufacturer’s instructions using RIPA buffer (Sigma-Aldrich) with the addition of complete protease inhibitor cocktail tablets (Roche). Protein concentrations were determined by nanodop. For immunoblot assays, liver proteins (50 µg/lane) in Lamelli buffer were separated by polyacrylamide gel electrophoresis and transferred to PVDF membranes. The membranes were blocked by 5% nonfat milk powder for 60 minutes and treated with primary antibody for one hour. Subsequently, the membranes were washed and incubated with anti rabbit IgG conjugate coupled with horseradish peroxidase for 1 hour. The membranes were again washed and the protein antibody complex detected using the ECL Advance Western Bot Dection Kit (GE healthcare). UQCR10 primary antibody (Abcam, Ab134909) was used according to manufacturer’s directions at a 1:10,000 dilution.

### Immunohistochemistry on paraffin embedded tissues

Five micron sections were de-paraffinized, rehydrated, and steamed in citrate buffer for 45 minutes. The slides were then incubated with the primary antibody for 1 hour at room temperature. For UQCR10 (Cat # 17779-1-AP, Proteintech) the antibody was used at a 1:100 dilution. The HBsAg (DAKO, Carpinteria, California, USA) is prediluted and was used neat. Following the primary antibody, the sections were incubated for 30 minutes in Dako EnVision + Peroxidase, a labeled-dextran polymer, followed by incubation with diaminobenzidine (DAB). Color development was monitored and stopped after 2–3 minutes.

### Immunohistochemistry on cells grown in cell culture wells

Cells were grown on glass slides until they were about 80% confluent. The medium was removed and the slides washed with 1 × PBS and fixed overnight in formalin and then washed with 1× PBS. Antigen retrieval was performed by steaming in sodium citrate buffer for 45 minutes and the cells were then permeabilized with Triton X-100 for 20 minutes, followed by blocking in hydrogen peroxide for 20 minutes. Primary antibody (UCRC Antibody, 17779-1-AP, Proteintech, dilution 1:100) or Dako (neat) for HBsAg was then added to the slides for 1 hour at room temperature followed by washing and the secondary HRP conjugated antibody incubated for 1 hour. After incubation with secondary antibody the slides were washed and detection performed using DAB substrate.

### ELISA

HBsAg and HBeAg ELISA assays (ETI-MAK-2 plus and ETI-EBK plus) were preformed on cell culture supernatant after centrifugation at 2,000 rpm for 5 minutes using the manufacturer’s protocol without modifications.

### DNA protection Assay

To determine whether viral DNA was protected from DNAse digestion, which would be anticipated if viral DNA is packaged into virions, supernatant from cell culture transfection experiments were studied. If the viral DNA is unpackaged, then it will be digested readily with DNAase. 48 hour supernatants were collected after temporary transfection of 1.3× vector-transgenic viral DNA constructs or from 1.0 transgenic viral DNA constructs. 100 ul of supernatant was digested with DNAase or was mock digested, with glycerol added instead of DNAase. After digestion, both the digested samples underwent DNA extraction (Qiagen) and real time PCR for viral DNA. If the viral DNA is packaged into a virion, the anticipated result is that there will be no difference in viral DNA levels by real time PCR between the mock and DNAse digested samples. If there is no protection of the viral DNA by a viral capsid, then a 2–3 Ct difference by real time PCR is anticipated, with the mock digested sample having more DNA than the DNAse digested sample.

### Strand bias PCR

*Overview*: Because of the unique replication strategy of HBV, packaged viral particles contain partially double stranded DNA. To determine whether the viral DNA in viral particles was in fact partially double stranded, we used strand-bias real time PCR. In this assay, three parallel PCR amplifications are performed: first round PCRs containing only the forward primer, only the reverse primer, or both primers. Next, second round PCR is performed using both forward and reverse primers and template from each of the three first round PCRs. If the HBV DNA is single stranded, there will be significant bias strand bias in the Cts between the PCR reactions.

1. DNA was extracted following the protection assay from samples that were positive, indicating the presence of viral coat or other structure protecting the DNA from digestion.
2. Perform real time PCR using 4 ul input of the DNA from step 1. Perform 15 rounds of PCR. Set up three separate PCR reactions. Primers are from Zanella *et al*.^7^.
  - Forward only primer (For4)
  - Reverse only primer (Rev7)
  - Both forward and reverse primers (For4 and Rev7).
3. Set up the second round of real time PCR using 2 ul DNA input from round 1. Set up a separate PCR reaction for each of the PCR reactions in step 2. The input for this step can be adjusted depending on the amount of viral DNA in the original extract. The goal is to have the 2.c PCR become positive in approximately the 12 to 20 Ct range.

### Flow cytometry

Flow cytometry was performed using the Flow Cytometry Core at Johns Hopkins School of Public Health. All samples were analyzed by the staff of the flow cytometry core using standard procedures.

#### Blocking studies

HepG2 cells were grown in cell culture inserts and temporarily transfected with transgenic HBV 1.3× constructs containing GFP. Separately, HepG2 cells with permanent expression of UQCR10 were seeded in 6 well plates and incubated with varying concentrations of Pre-S1 antibody (sc-57761, Santa Cruz Biotechnology). 24 hours after transfection of the cells in the inserts, the inserts were thoroughly washed and place in 6 well plates containing HepG2 cells with permanent expression of UQCR10 and varying concentrations of antibody. Cells were harvested for flow cytometry analysis at 37 and 72 hours of exposure to transgenic virus (produced by the cells in the inserts).

### Data availability

All data generated or analyzed during this study are included in this published article (and its supplementary information files). A detailed step by step protocol on the methods to create transgenic viruses are available as supplemental material.

## Electronic supplementary material

Supplemental data and protocols
