# Supplementary material for: Transgenic hepatitis B: a new model of HBV infection
Source: Sci Rep. 2017 Jun 1;7:2610. doi: 10.1038/s41598-017-02862-2 (PMC5453976; doi:10.1038/s41598-017-02862-2)
Supplement: Supplementary file 1 — Supplemental data and protocols [file 41598_2017_2862_MOESM1_ESM.pdf]

Transgenic hepatitis B: a new model of HBV infection

Hubert D-J Daniel, Ph.D.[1*], Michael Torbenson, M.D. [1*]

[1]Johns Hopkins University School of Medicine, Department of Pathology

*Now at CMC Vellore (DJD) and Mayo Clinic Rochester (MT)

Correspondence:
Michael S. Torbenson, M.D.
Professor of Laboratory Medicine and Anatomic Pathology
Phone: 507-284-3537
Secretary: 507-284-0697
Fax: 507-284-1599
Email: torbenson.michael@mayo.edu

Mayo Clinic
Hilton Building, 11th floor
200 First Street SW
Rochester, MN 55905

Section 1: Supplemental figures and tables
Section 2: Creation of transgenic HBV
Section 3. Main Experimental Assay

SECTION 1: SUPPLEMENTAL FIGURES AND TABLES

Supplemental Fig 1.

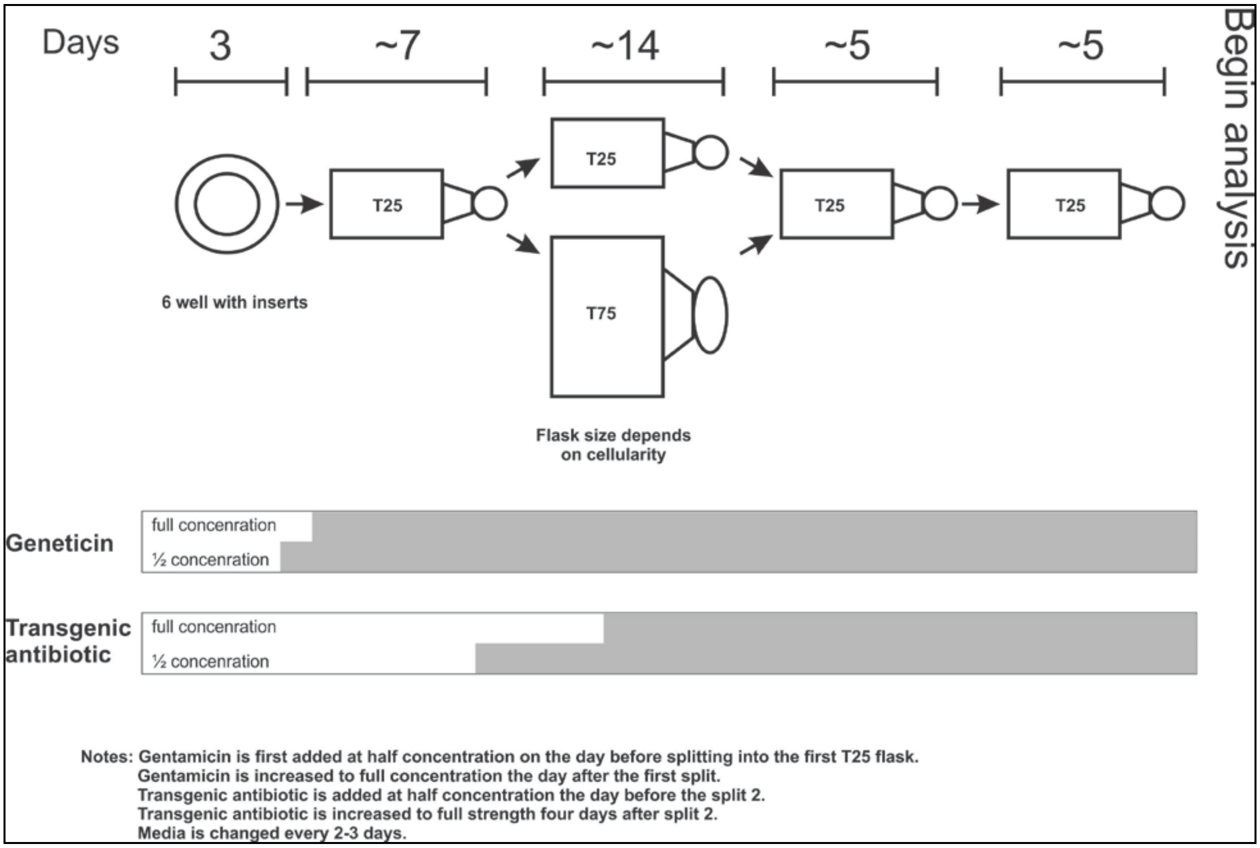

Supplemental Figure 1. Diagram of assay for missing receptor/replication factor.

Supplemental Figure 2

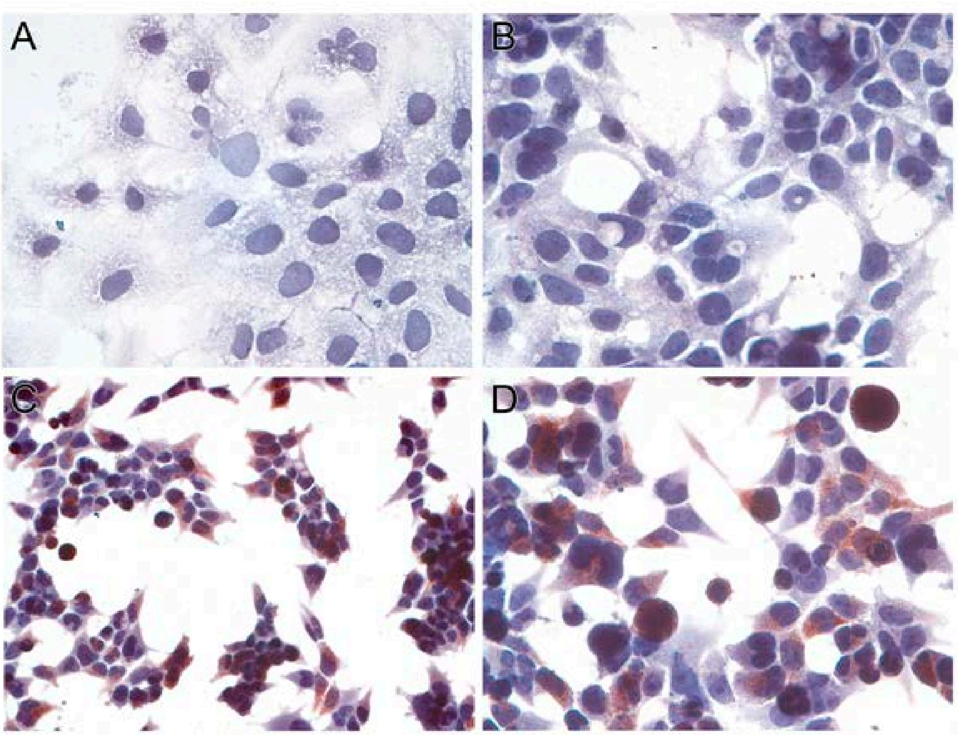

Supplemental Figure 2. Immunohistochemistry for hepatitis B surface antigen. Panel A (original magnification 260X). Wild type HepG2 cells are negative for HBsAg; Panel B (original magnification 260X). Wild type Huh7 cells are negative for HBsAg; Panel C (original magnification 160X). Fully selected HepG2 cells from the assay Sc20 are strongly positive for

HBsAg; Panel D (original magnification 260X). Higher magnification image from cells in panel C showing strong cytoplasmic staining.

Supplemental Figure 3.

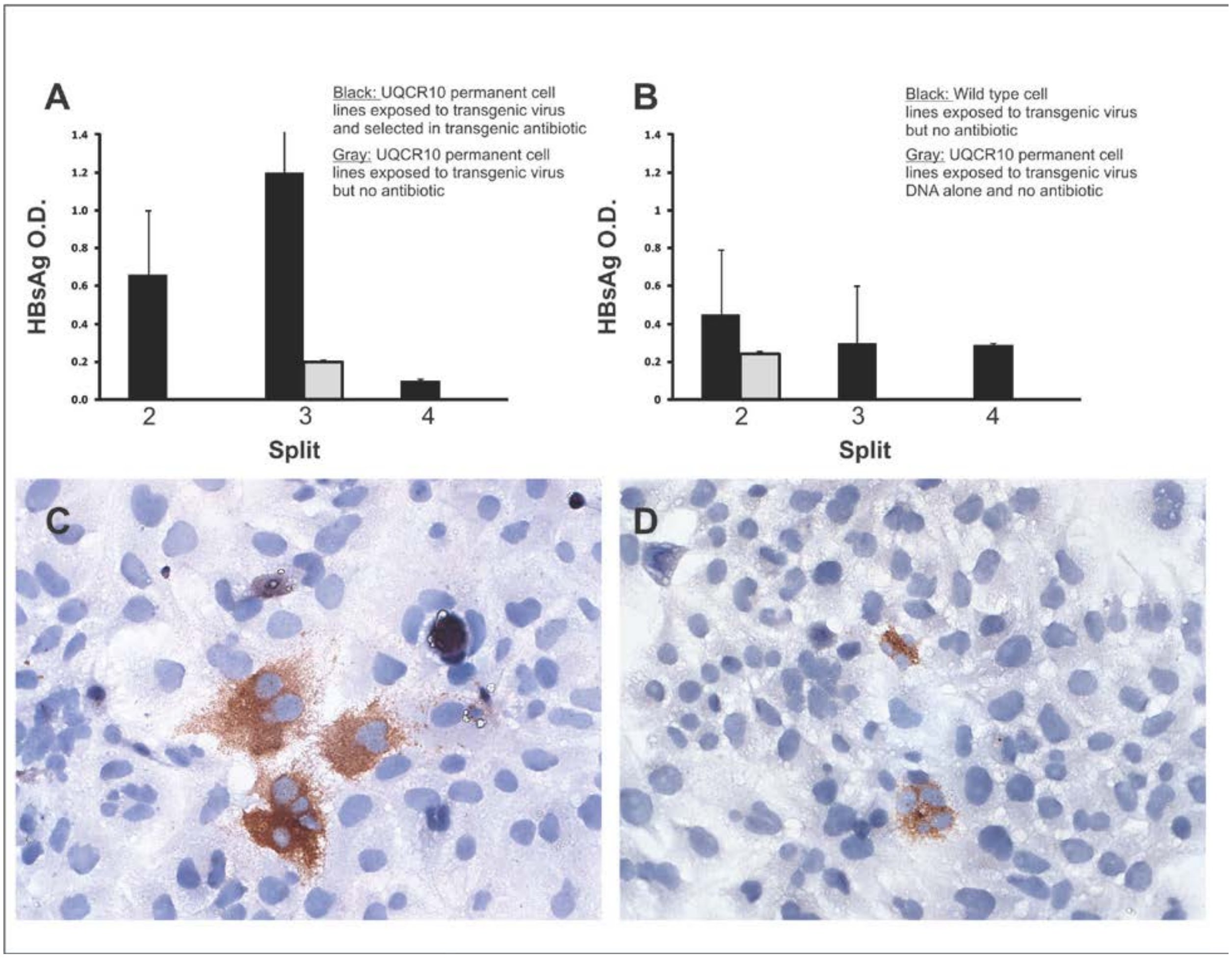

Supplemental Figure 3. HBsAg expression in the supernatants and cells from the experiments shown in figure 5. Panel A, Huh7 permanent UQCR10 cell line secretes more HBsAg into the supernatant when exposed to antibiotic pressure to retain the virus. Panel B, Transgenic HBV also enters wild type Huh7 cell lines. Panel C, UQCR10 permanent Huh7 cell lines after the 3rd split (panel A, black bar of this figure), show positive cytoplasmic staining for HBsAg. Panel D, The wild type Huh7 cells after the 3rd split (panel B, black bar of this figure) are also HBsAg positive.

Supplemental Figure 4.

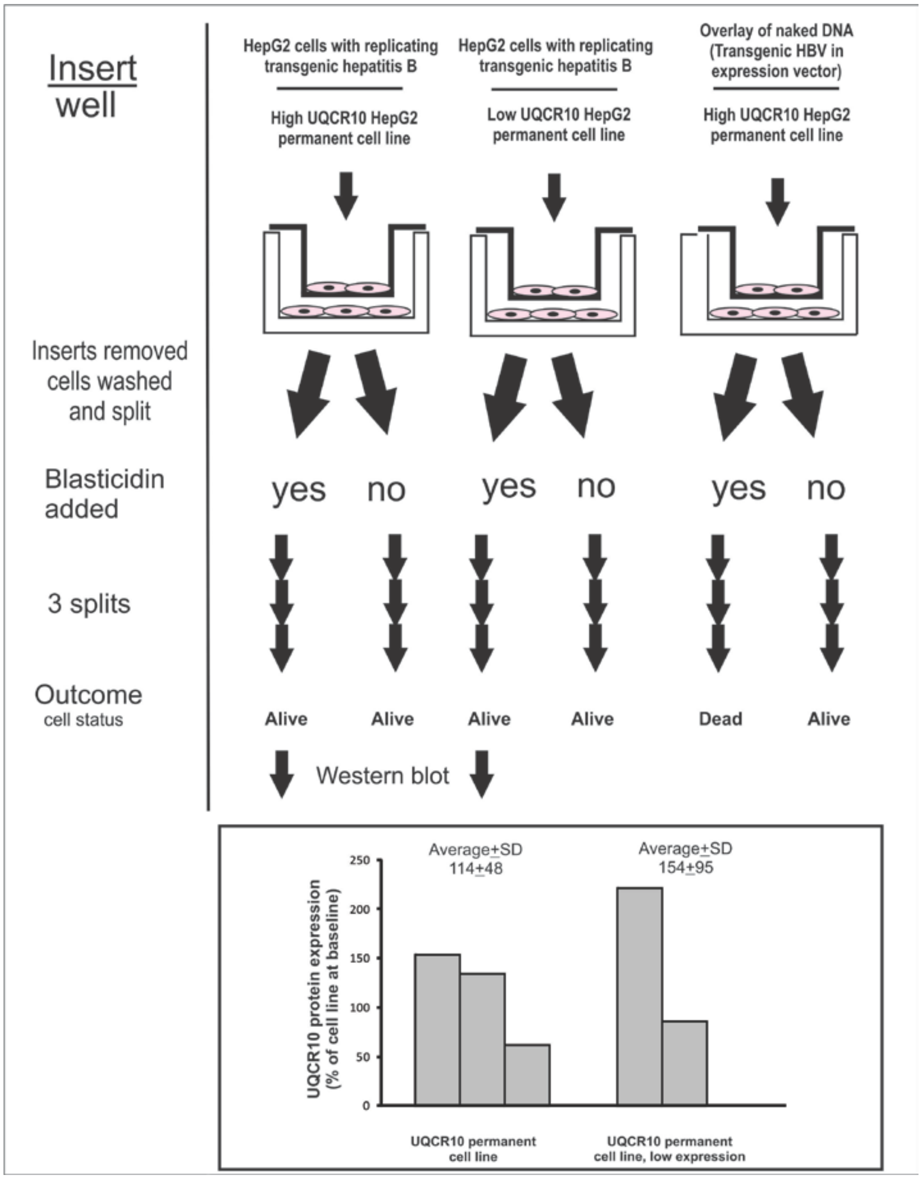

Supplemental Figure 4. Experimental design to investigate whether UQCR10 enhances sustained viral replication. A low-producing UQCR10 permanent cell line is exposed to transgenic virus. Permanent cell lines typically have a range of vector expression levels at the individual cell level. In this experiment, transgenic HBV can enter any cell within the low-producing UQCR10 permanent cell line, but when Blasticidin antibiotic pressure is added; those cell lines that can best support sustained viral replication are enriched. Western blots demonstrate that these cells have increased UQCR10 expression levels.

Supplemental Figure 5.

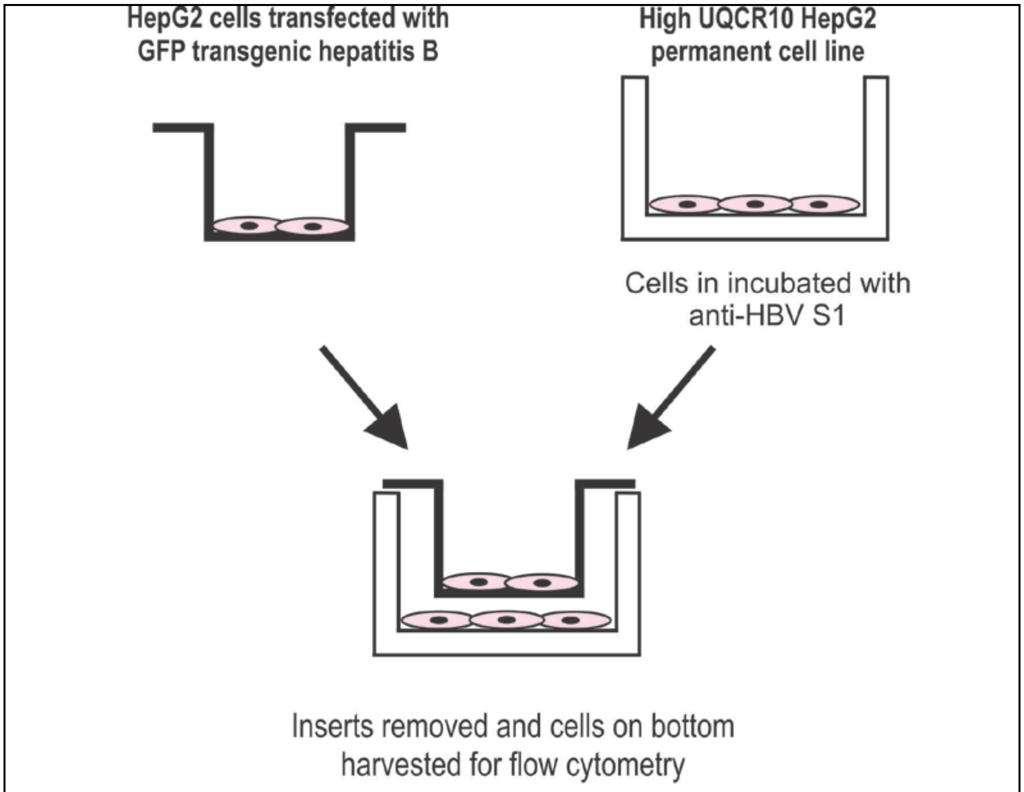

Average % GFP positive cells

Time	No antibody	10 ug antibody	50 ug antibody
36 hours	0.02$\pm$0.00	0.01$\pm$0.00	0.00$\pm$0.00
72 hours	9.4$\pm$1.9	4.9$\pm$1.0	1.79$\pm$0.41

Student t test, 96 hours, none vs 10 ug: p=0.02
Student t test, 96 hours, none vs 50 ug: p=0.002
Student t test, 96 hours, 10 ug vs 50 ug: p=0.007

Supplemental Figure 5. Hepatitis B binds to the cellular receptors via the viral pre-S1 surface antigen protein. This experiment demonstrates that transgenic HBV entry into UQCR10 permanent cell lines is dependent on pre-S1 protein because it can be blocked by pre-S1 antibody. Transgenic HBV encoding GFP were transfected into cells growing in the cell inserts. After 24 hours, the insert was placed into a well containing UQCR10 permanent cell HepG2 cells. After various times after exposure to transgenic virus (36 hours, 72 hours) the inserts were removed, cells were washed, and analyzed by flow cytometry.

Supplemental Figure 6.

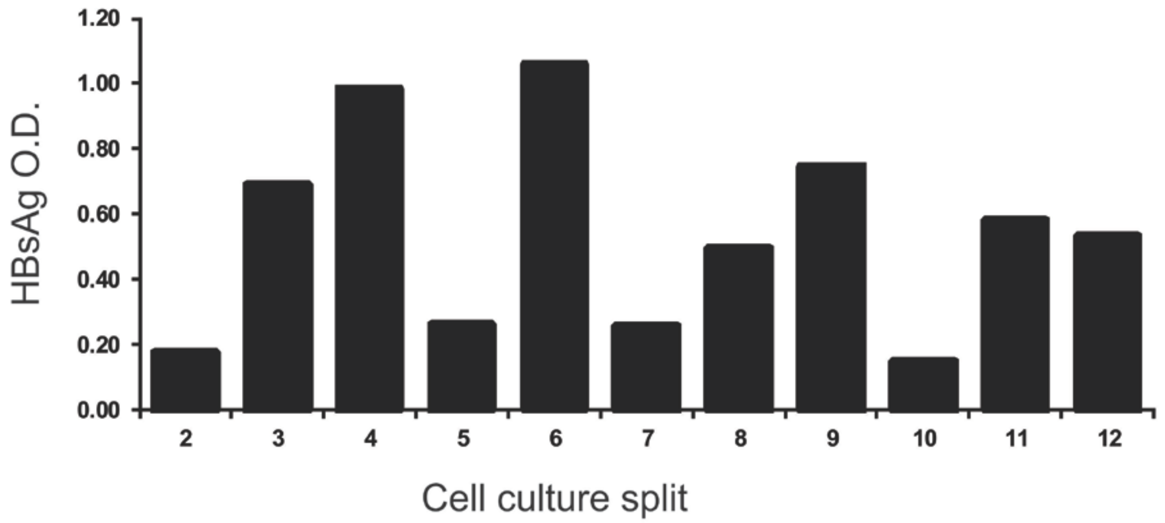

Split	2	3	4	5	6	7	8	9	10	11	12
average ± SD	0.17±0.1	0.69±0.8	0.98±0.9	0.26±0.2	1.06±1.0	0.26±0.2	0.49±0.7	0.74±0.58	0.15±0.08	0.58±0.75	0.53±0.38
range	0.07 - 0.31	0.07 - 2.6	0.1 - 2.6	0.07 - 0.51	0.21 - 2.5	0.07 - 0.71	0.09 - 3.3	0.23 -1.9	0.08 - 0.16	0.09 - 1.7	0.1 - 0.78
No. positive wells	11	11	9	13	8	12	10	11	6	5	3
Total No. wells	18	19	15	14	15	15	15	15	9	9	3

Supplemental Figure 6. Low but persistent HBsAg after exposure to human serum with unmodified HBV virions. Wild type HepG2 and UQCR10 permanent HepG2 cell lines were seeded in 6 well plates and exposed for 72 hours to 6 log copies of unmodified HBV virions from human serum pools. After this, cells were washed and split and HBsAg levels measured every 3-4 days, prior to splitting. Wild type control HepG2 cells (not shown) showed higher levels of HBsAg in the supernatant then UQCR10 for the first 2 splits, consistent with viral binding and slow release by the wild type cells, a finding also noted by others in other experimental models[19]. By split 5, controls were negative.

Supplemental Figure 7.

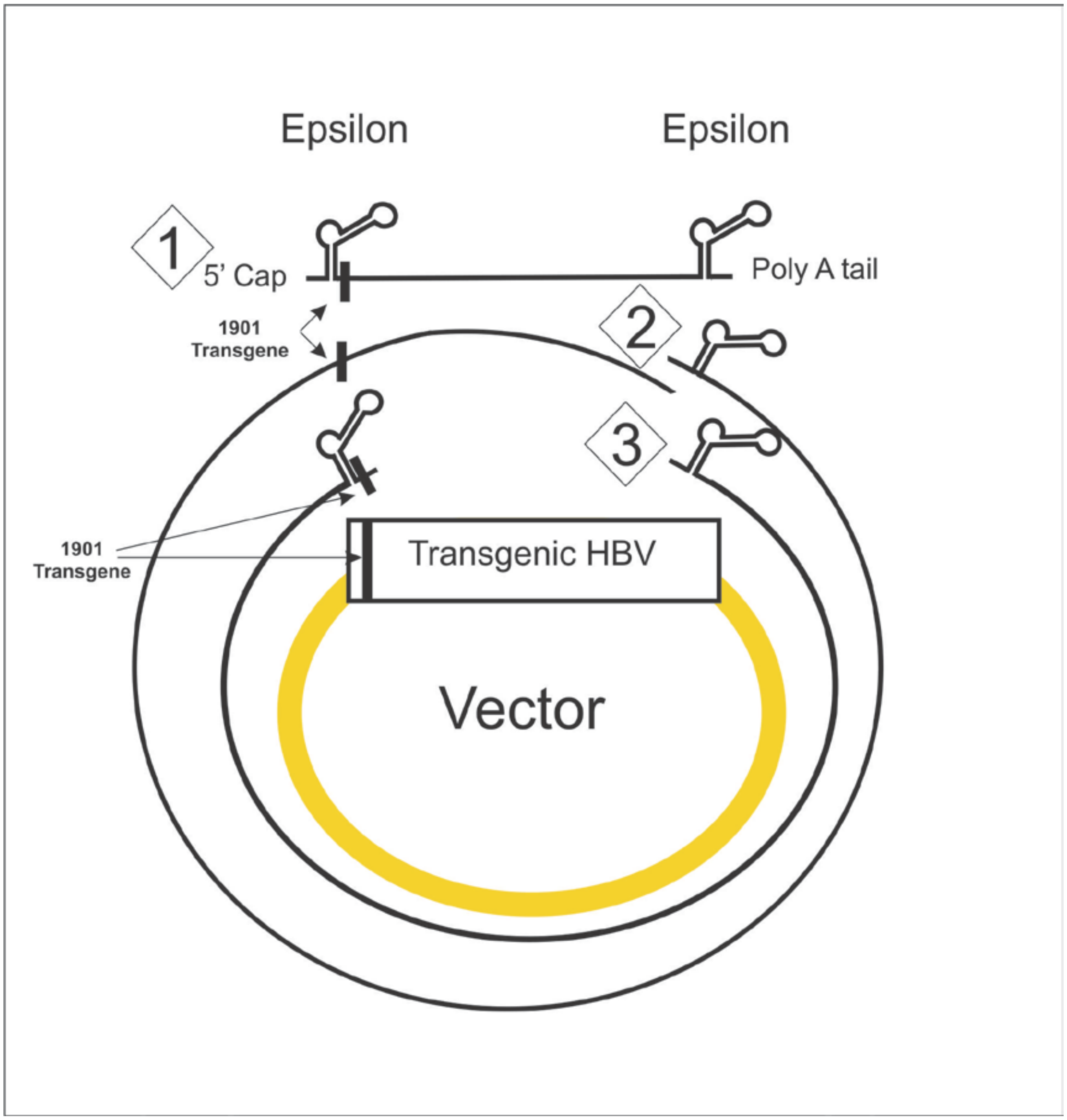

Supplemental Figure 7. pgRNA in transgenic virus/plasmid constructs. The transgenic viral-vector construct can serve as a template for multiple pgRNA transcripts. Because pgRNA is a greater than full length transcript of cccDNA, it has two terminal redundancies, which both contain a pgRNA start site as well as a polyA signal. The 1.3X length of transgenic HBV that is cloned into the vector serves the role of cccDNA and also has two terminal redundancies, which both contain a pgRNA start site as well as a polyA signal. Thus, multiple transcripts are possible. Transcript 1 (diamond) was the anticipated transcript given our HBV DNA construct and is the same size of the wild type pgRNA plus the transgene. Transcript 1 was directly detected using 5' race, which showed pgRNA transcripts that included the transgene in the appropriate location for transcript 1. Transcript 2 (diamond) encodes the entire transgenic virus

as well as the entire plasmid. For methodological reasons, transcript 2 is difficult to directly demonstrate but is inferred based on the results of the assays, including the negative controls that rule out nonspecific uptake of 1.3x transgenic HBV-plasmid constructs that might be released by dying cells in the inserts (Figure 2) or left over from transfection. Transcript 3 (diamond) encodes a “defective virus” that contains all of the vector and transgene but is missing most of the HBV DNA. This transcript was demonstrated by temporary transfection of the full 1.3X transgenic virus-plasmid constructs into HepG2 cells, followed by addition of Geneticin, which adds selective pressure to retain the Geneticin antibiotic resistance gene encoded by the vector. After several passages, cells were harvested, episomal DNA harvested by miniprep, and electroporated into bacteria. Clones that correlated with transcript 3 were easily found.

Supplemental Table 1. Transgenic hepatitis B produces both HBsAg and HBeAg 24 hours after transfection of 1.3X viral-plasmid constructs into HepG2 or Huh7 cell lines. Each transfection was preformed the same day and in duplicate. HepG2 cells generally produce more HBsAg while Huh7 cell lines generally produce more HBeAg. The 1901 Blasticidin and 1901 Zeocin produce the best HBsAg overall, and so where used in the main experimental assay for the missing viral entery/replication factor.

**Cell line**	**HBV construct**	**HBsAg O.D.**	**HBeAg O.D.**
HepG2	1852 Zeocin	0.66±0.04	0.21±0.01
HepG2	1852 GFP	0.93±0.13	0.19±0.03
HepG2	1901 Blasticidin	1.5±0.23	0.19±0.03
HepG2	1901 Zeocin	0.88±0.01	0.14±0.00
HepG2	1901 GFP	0.79±0.00	0.09±0.03
**Average**	**All transgenic constructs**	**0.95±0.32**	**0.16±0.05**
Huh7	1852 Zeocin	0.22±0.09	0.80±0.37
Huh7	1852 GFP	0.25±0.05	0.57±0.01
Huh7	1901 Blasticidin	0.39±0.08	0.62±0.14
Huh7	1901 Zeocin	0.30±0.00	0.44±0.20
Huh7	1901 GFP	0.21±0.02	0.29±0.00
**Average**	**All transgenic constructs**	**0.27±0.08**	**0.55±0.23**

Supplemental Table 2. Transgenic hepatitis B assay results.

Exp Name	Replic-ates	Cell line	Transgenic HBV/source of virus[1]	Outcome
Sc16	3,3	Huh7, HepG2	1901 blasticidin/ permanent	**Cells grow in 1/3 experiments each for Huh7 and HepG2; both are UQCR10 positive**
Sc16 control	3,3	Huh7, HepG2	Library transfection (+) Transgenic virus (-)	All cells die
Sc17	3,3	Huh7, HepG2	1901 zeocin/ permanent	**Cells grow in ?/3 experiments for HepG2 and are UQCR10 positive.** Remaining replicates die.
Sc17	3,3	Huh7, HepG2	1901 blasticidin / permanent	**Cells grow in 1/3 experiments for Huh7 and are UQCR10 positive.** Remaining replicates die.
Sc17	3,3	Huh7, HepG2	1901 zeocin/ transfection	All cells die
Sc17	3,3	Huh7, HepG2	1901 blasticidin / transfection	**Cells grow in ?/3 experiments for HepG2 and are UQCR10 positive.** Remaining replicates die.
Sc17 control	3,3	Huh7, HepG2	Library transfection (-) Transgenic virus (-)	All cells die
Sc17 control	3,3	Huh7, HepG2	Library transfection (+) Transgenic virus (-)	All cells die
Sc17 control	3,3	Huh7, HepG2	Library transfection (-) Transgenic virus (+)	All cells die
Sc17 control	3,3	Huh7, HepG2	Library transfection (-) Overlaid with extracted transgenic HBV-vector DNA (+)	All cells die
Sc19	3	HepG2	1901 zeocin/ transfection	**Cells grow in 1/3 replicates and are UQCR10 positive**
Sc19	3	HepG2	1901 zeocin/ blasticidin	**Cells grow in 1/3 replicates and are UQCR10 positive**
Sc19 control	3	HepG2	Library transfection (-) Transgenic virus (-)	All cells die
Sc19 control	3	HepG2	Library transfection (+) Transgenic virus (-)	All cells die
Sc19 control	3	HepG2	Library transfection (-) Transgenic virus (+)	All cells die
Sc19 control	3	HepG2	Library transfection (+) Overlaid with extracted transgenic HBV-vector DNA (+)	All cells die
Sc20	3	HepG2	1901 zeocin/ transfection	**Cells grow in 2/3 replicates and are UQCR10 positive**
Sc20	3	HepG2	1901 zeocin/ blasticidin	**Cells grow in 2/3 replicates and are UQCR10 positive**
Sc20 control	3	HepG2	Library transfection (-) Transgenic virus (-)	All cells die
Sc20 control	6	HepG2	Library transfection (+) Transgenic virus (-)	All cells die
Sc20 control	3	HepG2	Library transfection (-) Transgenic virus (+)	All cells die
Sc20 control	3	HepG2	Library transfection (+) Overlaid with extracted transgenic HBV-vector DNA (+)	All cells die

[1]Infectous transgenic hepatitis B virions were produced in the well inserts (see figure 1B) either by permanent transgenic hepatitis B cell lines, or by separately transfecting transgenic HBV DNA into the wild type cell lines 24 hours prior to starting the experiment.

Supplemental Table 3. Full coding of the UQCR10 gene from the expression vectors obtained at the end of the assay.

ATGGCGGCCGCGACGTTGACTTCGAAATTGTACTCCCTGCTGTTCCGCAGGACCTCCACCTTC
GCCCTCACCATCATCGTGGGCGTCATGTTCTTCGAGCGCGCCTTCGATCAAGGCGCGGACGC
TATCTACGACCACATCAACGAGGGGAAGCTGTGGAAACACATCAAGCACAAGTATGAGAAC
AAGTAGTTCCTTGGAGGCCCCCATCCAGGCCAGAAGGACCAGGTCCACCCAGCAGCTGTTTG
CCCAGAGCTGGAGCCTCAGCTTGAAGATGATGCTCAAGGTACTCTTCATGGACCACCATTCG
CTGTTGGCAAGAAACGGCTTTACTTACAAAACAGACTCTTTACCTTCTGCTGTGTTTGAAGTA
TGTTTAGTCAGCATGCTCAGGAAATAAGTGTGAATTGCCCTTGAAAAAAAAAAAAAAAAAA
A

Supplemental Table 4. UQCR10 mRNA levels were measured by real time PCR in cell lines and in fresh frozen human hepatocellular carcinoma tissues. The expression levels are normalized to normal human liver for the cell lines and to the paired non-neoplastic tissues for the human hepatocellular carcinomas.

**Cell line**	**Underlying liver disease**	**UQCR10 mRNA levels (fold change)**
HepG2	NA	11.7
Huh7	NA	6.2
HCC-1	Chronic hepatitis C	1.3
HCC-2	Chronic hepatitis C	4.2
HCC-3	Chronic hepatitis C	4.8
HCC-4	Chronic hepatitis C	2.1
HCC-5	Chronic hepatitis B	0.8

Supplemental Table 5. HBV DNA levels are maintained in the presence of antibiotic pressure, but are quickly lost if no pressure is applied. The antibiotic resistance gene for Blasticidin is encoded within the transgenic virus and adding the antibiotic encourages the cells to retain the virus. Data is mean and standard deviation for 3 replicates. HBeAg for split 4 is also shown.

	Huh7 permanent UQCR10 exposed to virus with antibiotic	Huh7 permanent UQCR10 exposed to virus but no antibiotic	Huh7 wild type exposed to virus but no antibiotic	Huh7 permanent UQCR10 exposed to viral DNA but no antibiotic
HBV DNA (copies per cell genome)				
Split 3	10.7±4.5	0.5±0.2	1.2±0.22	0.01±0.0
Split 4	5.8±0.5	0.03±0.2	0.2±0.16	0.01±0.0
HBsAg in supernatant (ELISA O.D.)				
Split 4	0.46±0.08	0.15±0.04	0.36±0.18	not detected
HBeAg in supernatant (ELISA O.D.)				
Split 4	0.08±0.01	not detected	0.03±0.02	not detected

Supplemental Table 6. Top 10 genes over and under expressed in both HepG2 and Huh7 permanent cell lines, normalized to their respective wild type cell lines.

**Cell line**	**Gene symbol**	Gene name	**Fold change**
Huh7 UQ up-regulated	S100A9	S100 calcium binding protein A9	7
	PLAC8	placenta-specific 8	6
	CYP1A1	cytochrome P450, family 1, subfamily A, polypeptide 1	6
	S100A4	S100 calcium binding protein A4	4
	INSIG1	insulin induced gene 1	4
	TESC	tescalcin	4
	LCN2	lipocalin 2	4
	DLK1	delta-like 1 homolog	3
	PFKFB4	6-phosphofructo-2-kinase/fructose-2,6-biphosphatase 4	3
	CD7	CD7 molecule	3
Huh7 UQ down regulated	EDN1	endothelin 1	-9
	TFF2	trefoil factor 2	-9
	FOS	FBJ murine osteosarcoma viral oncogene homolog	-6
	INHBE	inhibin, beta E	-6
	GDF15	growth differentiation factor 15	-6
	KLF2	Kruppel-like factor 2	-6
	FAM47E	family with sequence similarity 47, member E	-6
	DUSP1	dual specificity phosphatase 1	-6
	SERPINA3	serpin peptidase inhibitor, clade A (alpha-1 antiproteinase, antitrypsin), member 3	-5
	LRRC31	leucine rich repeat containing 31	-5
HepG2 up-regulated	AFP	alpha-fetoprotein	136
	SERPINA3	serpin peptidase inhibitor, clade A (alpha-1 antiproteinase, antitrypsin), member 3	113
	SPINK1	serine peptidase inhibitor, Kazal type 1	104
	AHSG	alpha-2-HS-glycoprotein	86
	APOB	apolipoprotein B	75
	A2M	alpha-2-macroglobulin	74
	GPX2	glutathione peroxidase 2	68
	PGC	progastricsin	66
	AGT	angiotensinogen	65
HepG2 down-regulated	TMSB15A	thymosin beta 15a	-16
	CD81	CD81 molecule	-14
	BASP1	brain abundant, membrane attached signal protein 1	-11
	ZCCHC12	zinc finger, CCHC domain containing 12	-11
	LDHB	lactate dehydrogenase B	-10
	FOXC1	forkhead box C1	-10
	BEX2	brain expressed X-linked 2	-8
	MLLT11	myeloid/lymphoid or mixed-lineage leukemia (trithorax homolog, Drosophila); translocated to, 11	-8
	VIM	vimentin	-8
	ADAMTS1	ADAM metallopeptidase with thrombospondin type 1 motif, 1	-7
			HepG2; Huh7
Top genes upregulated in both HepG2 and Huh7	APOM	apolipoprotein M	21; 2
	SLC16A3	solute carrier family 16, member 3	37; 3
	DLK1	delta-like 1 homolog	13; 3
	CD7	CD7 molecule	8; 3
	CRIP1	cysteine-rich protein 1	8; 2
	SULT2A1	sulfotransferase family, cytosolic, 2A, dehydroepiandrosterone	8; 3

		(DHEA)-preferring, member 1	
	UBD	ubiquitin D	7; 2
	HMGCS1	3-hydroxy-3-methylglutaryl-CoA synthase 1	5; 2
	CRLF1	cytokine receptor-like factor 1	5; 2
	TESC	tescalcin	5; 4
			HepG2; Huh7
Top genes down regulated in both HepG2 and Huh7	ADM	adrenomedullin	-2; -3
	ANXA1	annexin A1	-2; -3
	DHRS7	dehydrogenase/reductase (SDR family) member 7	-2; -2
	DMKN	dermokine	-2; -3
	F12	coagulation factor XII (Hageman factor)	-2; -3
	HIST1H2BK	histone cluster 1, H2bk	-2; -2
	PPP1R15A	tein phosphatase 1, regulatory subunit 15A	-2; -4
	PRSS23	protease, serine, 23	-2; -2
	RBP1	retinol binding protein 1, cellular	-3; -2
	SIPA1L2	signal-induced proliferation-associated 1 like 2	-3; -2

Supplemental Table 7. mRNA expression levels of genes associated with hepatocyte maturation were measured by real time PCR. Fold change in mRNA (Log2) is shown. The UQCR10 permanent cell lines were normalized to their paired wild type cell lines.

**Cell line**	**Albumin**	**HNF4a**	**FOXA2**	**PXR**	**GATA4**	**Ahr**
HepG2-UQCR10	-11.0	-10.0	-3.5	-3.9	-0.7	-1.7
Huh7- UQCR10	0.0	1.7	1.4	not detected	0.9	3.0

Supplemental Table 8. Functional studies of the effect of UQCR10 on cell lines. Each experiment was performed in duplicate, with two replicates each. The HBsAg levels were measured 24 hours after transfection.

**Cell line**	HepG2 Wild type	HepG2-UQCR10	Huh7 Wild type	Huh7- UQCR10
ATP production (nMol) Mean±SD	5.1± 0.2	4.5±0.2	3.8±0.2	3.8±0.1
Proliferation (O.D) Mean±SD	2.0± 0.3	1.6± 0.1	2.7± 0.3	2.0± 0.5
Phagocytosis (O.D) Mean±SD	0.8±0.2	0.92±0.3	1.6±0.1	1.3±0.2
Supernatanant HBsAg 1852/Zeocin (0.D), Mean±SD	0.8±0.1	3.0±0.3	0.5±0.2	0.3±0.1
Supernatanant HBsAg 1901/Zeocin (0.D), Mean±SD	2.8±0.9	6.6±0.3	0.6±0.4	0.4±0.0
Supernatanant HBsAg 1901/blasticidin (0.D), Mean±SD	3.4±0.3	6.5±0.3	0.4±0.1	0.3±0.1

Supplemental Table 9. Characterization of cell culture supernatant for cell lines with permanent 1.0 transgenic viral cccDNA. Each experiment was performed in duplicate on wild type cells that underwent temporary transfection, were split after 4 days, and supernatant collected after another 4 days.

Cell line	1.0 transgenic ccc DNA	HBsAg (O.D)	HBeAg (O.D)
HepG2	1852 Zeocin	0.6±0.2	0.2±0.0
Huh7	1852 Zeocin	0.6±0.1	0.2±0.0
HepG2	1901 blasticidin	0.2±0.0	Not detected
Huh7	1901 blasticidin	0.2±0.0	Not detected

SECTION 2: CREATION OF TRANSGENIC HBV

Overview. Insertion of a foreign gene into the HBV genome is challenging without disrupting viral gene expression because the viral genes are coded in overlapping open reading frames and there are no introns. Guided by the genetic map of HBV, we created transgenic viruses by inserting transgenes at sites 1852 and 1901 (all numbering is from traditional ECOR1 digestion site). Of note, both the 1852 and 1901 sites are at ATG sites in the HBV genome, but they are not known to be the start site for mRNA transcripts that are transcribed into proteins, though possible native viral RNA transcripts starting at one these sites have been reported.[1]

Careful consideration of the known HBV genetic map shows the following: (1) insertion of new genes at 1852 and 1901 sites do not interrupt the P, S, X, or C open reading frames; (2) DR1 and DR2 are not affected and the epsilon region is not affected (1901 insertion site) or affected only at the outer edge of the Epsilon coding region (1852 insertion site). Epsilon is a segment of the pgRNA that forms a hairpin secondary structure that is necessary for pgRNA encapsidation and for DNA replication; (3) pgRNA is affected and increases in length because of the transgene; (4) the HBeAg is affected by both insertion sites, but for the 1852 insertion site, the affected part is normally cleaved off in the Golgi prior to secretion of HBeAg. Both insertion sites secrete HBeAg, but the 1901 insertion site secretes HBeAg at a reduced level.

In order to create packaged transgenic virus, we constructed greater-than full length virus (1.3X full length) that contained the transgene and cloned them into an expression vector with a CMV promoter (pcDNA3.1/V5-His TOPO TA Expression kit, Invitrogen). This 1.3X clone of HBV DNA serves the function of cccDNA in the permanent cell line.

Careful study of the HBV life cycle indicated two different ways to create transgenic HBV constructs. In both cases, transgenes are inserted in the R regions, which are located at the 5’

and 3’ end of pgRNA. In the first construct, the transgene is inserted in the 5’ R, while in the second, the transgenes is inserted in the 3’R (Figure 1 A, B). These designs have very different theoretical outcomes: the first construct produces fully packaged transgenic HBV, while the second construct should package only wild type virus. In the second model, the transgene is in the 3’ R region and is included in the pgRNA but not in the fully reverse transcribed virus.

CREATION OF TRANGENIC VECTOR

Source of HBV DNA: A full length genotype D HBV genome was used to create the transgenic viruses. The internal laboratory designation for this cloned HBV DNA is HR90. The HBV genome was isolated from the de-identified serum of an individual who was HBeAg positive and had a high viral load (7.4 log HBV DNA /ml). We have used this same full length virus in previous studies investigating the role of methylation in viral replication.[2-4] Thus, we have experience with transfection of the full length wild type virus into HepG2 and Huh7 cells and the subsequent replication patterns, viral protein production patterns, and viral protein secretion patterns of this wild type virus.

The HBV virus was originally amplified with P1 and P2 primers[5] and cloned into a cloning vector (pSC-A, stratagene). Two clones were selected, validated, and stored and are used in subsequent studies.[2-4] They are called HR90 clone 3 and HR90 clone 4. They have minor differences in their nucleotide sequence. HR90 clone 4 was used for these studies.

Transgenes used:
1. Zeocin (source: pDONR/Zeo, Invitrogen)
2. Blasticidin (source: pcDNA6.2/V5-DEST, Invitrogen)
3. Enhanced GFP (source: pRSET/EmGFP, Invitrogen)
4. CMV promoter (source: pcDNA3.1/V5-His TOPO TA Expression kit, Invitrogen)

Expression vector used to clone in 1.3X transgenic HBV
Invitrogen, pcDNA3.1/V5-His TOPO TA Expression kit

Transgenic HBV DNA constructs that were created
Transgenic HBV with the transgene in the forward position
1. HR90, 1852, Zeocin
2. HR90, 1852, GFP
3. HR90, 1901, Zeocin
4. HR90, 1901, Blasticidin
5. HR90, 1901, GFP

Transgenic HBV with the transgene in the back position
1. HR90, 1852, Zeocin
2. HR90, 1852, GFP
3. HR90, 1901, Zeocin
4. HR90, 1901, Blasticidin
5. HR90, 1901, GFP

Transgenic HBV with the transgenes in both locatons
1. HR90, 1901 CMV promoter; 1852 GFP

PROTOCOL TO PLACE TRANSGENE IN FORWARD POSITION

Overview. The transgenic HBV is constructed in four segments.

	Segment 1	Segment 2 (transgene)	Segment 3	Segment 4
Sticky ends	BspQI	BspQI	BspEI 4	

Segment 1: from 1227 to 1901 or 1852; approximate size: 674 bp (1901) or 625 bp (1852)
Segment 2: Transgenes; size varies by gene length
Blasticidin: 399 bp
Zeocin: 375 bp
GFP: 720 bp
CMV promoter: 652 bp
Segment 3: from 1901 or 1852 to 2329; approximate size: 430 bp (1901) or 480 bp (1852)
Segment 4: from 2333 to 2111 approximate size: 3100 bp
Total length: approximately 4.3 kb of HBV DNA

A. Form circular HBV DNA to use as templates for PCR

1. Grow vector with HR90 clone 4 in bacteria. HR90 is the full length HBV DNA amplified using "*P1*" and "*P2*" primers and cloned into a cloning vector.
2. Do a miniprep (Qiagen).
3. Digest the HBV DNA out using **BspQI (NEB, R0712)**
4. Gel purify the full length HBV DNA. Do not use eithidium bromide or UV on the actual sample.

   Note: To do this, run in parallel another lane. Cut this lane out of the gel and stain with eithidium bromide and visualize with UV. Notch the band that you see on UV with a razor blade. Next, put the gel back together and your notch will then tell you where your band is on the unstained gel. This method should always be used when there is a need to gel purify.
5. Check the DNA quality and quantity of the band (eg check gel, real time PCR, nanodrop).
6. Ligate the two ends of the HBV DNA to form circular DNA. Use T4 DNA ligase (Invitrogen).

B. Create segment 1

7. Perform PCR for 25 cycles using pfu polymerase, DNA from step No. 6, and primers "*island 2 cut and paste 1F*" and "*HBV TG 1852 TG R*" (or "*island 2 cut and paste 1F*" and "*HBV TG 1901 R*").

   Note: For all PCR steps throughout this protocol, pfu polymerase should be used and PCR should be limited to 25 cycles.
8. Gel purify. Alternatively, this can be cloned

C. Create the transgene.

9. Using the *transgene specific primers* (Table 1), PCR with pfu and 25 cycles of PCR, using vector as template. Gel purify as above.

10. Clone this into a cloning vector. Sequence and confirm.
11. Do a miniprep and purify the DNA. Check DNA quantity and quality.
12. Digest out the transgene DNA with **BspQI (NEB, R0712)** and gel purify.

    Note: An alternative is to amplify for 25 cycles with pfu using primers from step 9. Still need to gel purify and digest.

D. Ligate segment 1 to the transgene (Segment 2).

13. Digest the DNA from step 8 (segment 1) with **BspQI (NEB, R0712).** Gel purify.
14. Ligate the DNA fragments from steps 12 (transgene) and 13 (segment 1) with T4 DNA ligase.
15. Gel purify the ligated DNA without exposure to ethidium bromide or UV.
16. Clone the DNA from step 15. Use this as template to re-amplify using "*island 2 cut and paste 1F*" (same primer as in step 7) and the *transgene specific R* primer.
17. Digest the PCR product DNA from step 16 with **BspQI (NEB, R0712)** and gel purify without exposure to ethidium bromide and UV.

E. Create and ligate segment 3.

18. Amplify segment 3 using DNA from step No. 6 as template and the primers "*HBV TG1852F*" or "*HBV TG1901 1F*" as forward primers and "*transgenic IS2 2R*" as the reverse primer.
19. Digest DNA from step 18 with **BspQI (NEB, R0712)** and gel purify without exposure to ethidium bromide and UV.
20. Ligate the DNA from steps 17 and 19.
21. Clone the ligated product (you should now have segments 1, 2 and 3 all ligated in the proper orientation).
22. Using DNA from step 21 as template (or alternatively, DNA from step 20), re-amplify using pfu, 25 cycles of PCR and primers "*island 2 cut and paste 1F*" and "*transgenic IS2 2R*". Digest with **BspEI (NEB, R0540L)**. Gel purify the larger fragment (enzyme cuts at bp 2329; the smaller fragment that you will discard should be approximately 450 bp)

F. Create segment 4 and ligate to create the full 1.3X length transgenic HBV

23. Use "*HBV 2111 Sap1 F*" and "*HBV 2111 Sap1 R*" to amplify a full length HBV DNA fragment using the DNA from step No.6 with pfu and 25 cycles of PCR. Clone into a cloning vector.
24. Digest the DNA from step 24 using with **BspEI (NEB, R0540L)**. This will give a 3.1 kb product and a 222 bp product. Gel purify the 3.1 kb product.

    Note: Instead of using cloned DNA, an alternative is to use the PCR product from step 23, digest, and gel purify.
25. Ligate DNAs from step 22 and 24.
26. Clone into expression vector (pcDNA3.1/V5-His TOPO TA Expression kit, Invitrogen).
26. Select clones and verify insert size. Sequence.

PROTOCOL TO PLACE TRANSGENE IN BACK POSITION

Overview. The transgenic HBV is constructed in four segments.

Segment 1	Segment 2	Segment 3 (transgene)	Segment 4

Sticky ends: SphI BspQI BspQI

Segment 1: from 1527 to 1244; approximate size: 2967 bp
Segment 2: from 1227 to 1901 or 1852; approximate size: 675 bp (1901) or 625 bp (1852)
Segment 3: Transgenes; size varies by gene length

Blasticidin: 399 bp
Zeocin: 375 bp
GFP: 720 bp
CMV promoter: 652 bp

Segment 4: 1901 or 1852 to 2950: approximate size: 1098bp (1852) or 1049bp (1901)
Total length: approximately 5.3 kb

<u>A. Form circular HBV DNA to use as templates for PCR</u>

1. Grow vector with HR90 clone 4 in bacteria. HR90 is the full length HBV DNA amplified using "*P1*" and "*P2*" primers and cloned into a cloning vector.
2. Do a miniprep (Qiagen).
3. Digest the HBV DNA out using **BspQI (NEB, R0712)**
4. Gel purify the full length HBV DNA. Do not use eithidium bromide or UV on the actual sample.

   Note: To do this, run in parallel another lane. Cut this lane out of the gel and stain with eithidium bromide and visualize with UV. Notch the band that you see on UV with a razor blade. Next, put the gel back together and your notch will then tell you where your band is on the unstained gel. This method should always be used when there is a need to gel purify.
5. Check the DNA quality and quantity of the band (eg check gel, real time PCR, nanodrop).
6. Ligate the two ends of the HBV DNA to form circular DNA. Use T4 DNA ligase (Invitrogen).

<u>B. Create segment 1</u>

7. Use primers "*Transgenic IS2 1F*" and "*LP2 cut and paste 1R*" to amplify a HBV DNA fragment using the DNA from No. 6 above.
8. Gel purify without UV or ethidium bromide. Clone if desired.

<u>C. Create the transgene.</u>

9. Using the *transgene specific primers* (Table 2), PCR with pfu and 25 cycles of PCR, using vector as template. Gel purify as above.
10. Clone this into a cloning vector. Sequence and confirm.
11. Do a miniprep and purify the DNA. Check DNA quantity and quality.
12. Digest out the transgene DNA with **BspQI (NEB, R0712)** and gel purify.

Note: An alternative is to amplify for 25 cycles with pfu using primers from step 9. Still need to gel purify and digest.

D. Ligate segment 2 to the transgene (Segment 3).

13. Amplify segment 2 using pfu, 25 cycles PCR, DNA from No.6 as template and the primers "*IS2 cut and paste 1F"* with *"HBVTG 1901 R"* or *"HBVTG 1852 R"*.
14. Gel purify without UV or ethidium bromide. Alternatively, this can be cloned.
15. Digest the DNA from step 12 (transgene, segment 2) and step 14 (segment 3) with **BspQI (NEB, R0712).**
16. Ligate the DNA fragments from step 15 with T4 DNA ligase.
17. Gel purify the ligated DNA without exposure to ethidium bromide or UV.
18. Clone the DNA from step 17. Use this as template to re-amplify using *"IS2 cut and paste 1F"* and the *transgene specific R* primer. You should now have segments 2 and 3 joined.

E. Create and ligate segment 4.

19. Amplify segment 4 using pfu, 25 cycles PCR, DNA from No.6 as template and primers "*HBV TG1852F"* or "*HBV TG1901 1F"* as forward primers and "*transgenic IS2 PstI 1R*" as the reverse primer.
20. Separately digest DNA from steps 18 and 19 with **BspQI (NEB, R0712)** and gel purify without exposure to ethidium bromide and UV.
21. Ligate the DNA from step 20.
22. Clone the ligated product (you should now have segments 2, 3 and 4 ligated in the proper orientation).
23. Use this as template to re-amplify using "*IS2 cut and paste 1F"* and the "*transgenic IS2 PstI 1R"* primer.

F. Ligate segment 1

24. Digest the PCR product DNA from step 23 with **SphI (NEB, R0182S)** and gel purify without exposure to ethidium bromide and UV.
25. Digest DNA from step 8 with **SphI (NEB, R0182S)** and gel purify without exposure to ethidium bromide and UV.
26. Ligate DNA from steps 24 and 25.
27. Clone into expression vector (pcDNA3.1/V5-His TOPO TA Expression kit, Invitrogen).
28. Select clones and verify insert size. Sequence.

Note: If there is problems cloning, primer "*Transgenic island 2 EcoRI 1F*" can also be use (same primer as *Transgenic IS2 1F"* but with a digestion site to help in cloning.

Section 1, Table 1. Primers used in HBV transgenic constructs with transgene in the forward position.

**Primer name**	**Primer sequence, 5' to 3'**	**HBV Bp location**
P1 [5]	CCGGA AAGCTT GAGCTCTTC TTTTTCACCTCTGCCTAATCA	1821
P2 [5]	CCGGA AAGCTT GAGCTCTTC AAAAAGTTGCATGGTGCTGG	1821
HBV TG1852F	ccggaaagcttgagctcttctATGTCCTACTGTTCAAGCCTCCAAGC	1852
HBV TG1852R	ccggaaagcttgagctcttccCATGAACATGAGATGATTAGGCAGAG	1852
HBV TG1901 1F	ccggaaagcttgagctcttctATGGACATTGACCCTTATAAAGAATTTGG	1901
HBV TG1901 1R	ccggaaagcttgagctcttccCATGCCCCAAAGCCACCCAAGGCACAGC	1901
zeo 1F	ccggaaagcttgagctcttccATGGCCAAGTTGACCAGTGCCG	
zeo 1R	ccggaaagcttgagctcttcacatTCAGTCCTGCTCCTCGGCCACG	
blasto 1F	ccggaaagcttgagctcttccATGGCCAAGCCTTTGTCTCAAGAAG	
blasto 1R	ccggaaagcttgagctcttcacatTTAGCCCTCCCACACATAACCAGAG	
CMV promoter 1F	ccggaaagcttgagctcttccATGTACGGGCCAGATATACG	
CMV promoter 1R	ccggaaagcttgagctcttcacatAATTTCGATAAGCCAGTAAGCA	
enhanced GFP 1F	ccggaaagcttgagctcttccATGGTGAGCAAGGGCGAGGAG	
enhanced GFP 1R	ccggaaagcttgagctcttcacatTTACTTGTACAGCTCGTCCAT	
island 2 cut and paste 1F	TCAGCgcatgcGTGGAAC	1227
Transgenic IS2 2R	TACCCGCCTTCCATAGAGTG	2778
HBV 2111 Sap1 F	CCGGAAAGCTTGAGCTCTTC TGGGTGGGAGTTAATTTGGA	2111
HBV 2111 Sap1 R	CCGGAAAGCTTGAGCTCTTC ACCCAGGTAGCTAGAGTC	2111

Section 1, Table 2. Primers used in HBV transgenic constructs with transgene in the back position.

**Primer name**	**Primer sequence, 5' to 3'**	**HBV Bp location**
P1 [5]	CCGGA AAGCTT GAGCTCTTC TTTTTCACCTCTGCCTAATCA	1821
P2 [5]	CCGGA AAGCTT GAGCTCTTC AAAAAGTTGCATGGTGCTGG	1821
Transgenic IS2 1F	CACCTCTCTTTACACGGACT	1527
Transgenic island 2 EcoRI 1F	GATGAATTC CACCTCTCTTTACGCGGACT	1527
LP2 cut and paste 1R	GTTCCACgcatgcGCTGA	1244
Transgenic island 2 PstI 1R	GCTGCAGCAGGTGTCCTTGTTGGGATT	2950
zeo 1F	ccggaaagcttgagctcttccATGGCCAAGTTGACCAGTGCCG	
zeo 1R	ccggaaagcttgagctcttcacatTCAGTCCTGCTCCTCGGCCACG	
blasto 1F	ccggaaagcttgagctcttccATGGCCAAGCCTTTGTCTCAAGAAG	
blasto 1R	ccggaaagcttgagctcttcacatTTAGCCCTCCCACACATAACCAGAG	
CMV promoter 1F	ccggaaagcttgagctcttccATGTACGGGCCAGATATACG	
CMV promoter 1R	ccggaaagcttgagctcttcacatAATTTCGATAAGCCAGTAAGCA	
enhanced GFP 1F	ccggaaagcttgagctcttccATGGTGAGCAAGGGCGAGGAG	
enhanced GFP 1R	ccggaaagcttgagctcttcacatTTACTTGTACAGCTCGTCCAT	
HBV TG1852F	ccggaaagcttgagctcttctATGTCCTACTGTTCAAGCCTCCAAGC	1852
HBV TG1852R	ccggaaagcttgagctcttccCATGAACATGAGATGATTAGGCAGAG	1852
HBV TG1901 1F	ccggaaagcttgagctcttctATGGACATTGACCCTTATAAAGAATTTGG	1901
HBV TG1901 1R	ccggaaagcttgagctcttccCATGCCCCAAAGCCACCCAAGGCACAGC	1901

SECTDION 3. ASSAY TO IDENTIFY ENTRY/ REPLICATION FACTORS

Overview The main experimental assay (outlined in Supplemental Figure 1) is a functional assay based on the premise that HepG2 and Huh7 cells lack a molecule necessary for viral entry/ persistent replication. If this molecule is replaced, then the cells should be able to support natural infection by intact HBV virions. Relevant to this, there is strong scientific evidence that HBV virions can be produced once HBV DNA gets into the nucleus, for example by transfection.

In order to have the cell lines produce the molecule necessary for HBV viral entry/replication etc, an expression library was made from normal human liver tissue; the library is anticipated to express all mRNA in the normal liver, including the missing molecule. After transfection of this library into the HepG2 and Huh7 cell lines, some fraction of the cells will now express the missing molecule necessary for viral entry/persistent post-entry replication—but how to identify which cells have this missing molecule?

We decided on a functional approach wherein the final assay "signal' would be virus that entered and replicated within the cell lines, allowing the cells to be identified either by flow cytometry or by selection in antibiotic. A functional approach has the strong advantage of being able to identify the molecule regardless of whether it is part of the receptor or needed for persistent post-entry replication of the virus.

This approach has several particular strengths. *First*, expression libraries can have a bias towards smaller genes; thus, if the receptor or replication factor is large, it may not be well represented in the library. However this risk is mitigated by our approach which identifies both the receptor/persistent replication factors as well as any protein that upregulates these molecules.

Second, the assay is not a binding assay but instead is a functional assay; by its very nature, our assay identifies only those candidates that lead, in the end, to the complete cycle of viral entry and persistent viral replication. This gives our assay substantial power to screen out false positives—proteins that may bind nonspecifically to the viral proteins but are not the true receptors/replication factors. *Third,* this assay can be easily used in a variety of cell lines. This provides greater assurance of a positive result when two different cell lines lead to the same molecule or molecules in a shared pathway.

Outline of the experimental assay. Wild type HepG2 and Huh7 cell lines are first transfected with a liver expression library in 6 well plates (Figure 2 and supplemental figure 1). A small proportion of these cells will now express the molecule(s) necessary to support viral infection.

Next, cell culture well inserts are placed into the well and these inserts contain huh7 cells or HepG2 expressing the transgenic virus. There are now two cell populations that share the media, but they are physically separated. The bottom cell population is expressing an unknown molecule that is needed for viral infection, while the other cell population, growing in the insert, is secreting transgenic virus.

After 3 days of co-culture with the inserts, the inserts are removed and antibiotic added to the cell culture media. Separate antibiotics are added for both the library (Geneticin)—to ensure that the expression vector containing the receptor or replication factor is retained—and either Zeocin or Blasticidin to select those cells that have taken up HBV. Only those cells that are able to pick up the HBV virions and have persistent viral replication and have a library expression vector can survive.

After a clone of stable cells has grown in the presence of antibiotics (this takes many weeks because, as expected, there are <1% of cells that survive the initial selection), the cells are harvested, DNA extracted, and PCR performed with primers directed against the insert in the library expression vector.

As a second approach for analysis of cells cells selected out by the assay, there is a somewhat narrow window during the selection process where the cells are fully selected but the vectors have not yet become integrated into the hepatocyte genomic DNA (eventually all vectors used in this study will likely become integrated when under antibiotic selection pressure). We take cells during this window period, perform a modified mini-prep to isolate the plasmids,[6] and then transform bacteria and identify genes by routine colony selection and sequencing.

PROTOCOLS

Normal liver library. An expression library was made using de-identified fresh frozen liver tissue from a 35 year old woman with no underlying liver disease (liver resection for benign liver tumor).

1. mRNA was isolated (MAG mRNA Isolation, Invitrogen) and a cDNA library constructed as per the manufacturer's instructions.
2. The library was then transferred to an expression vector (pT-REex-Dest30, Invitrogen), as per the manufacturer's instructions.
3. The library was validated at every step as per the manufacturer's protocol. The average size insert was 1.4 Kb, range 0.6 to 4.2 Kb, based on sizing of 25 random clones.
4. A random sequencing of clones revealed genes typical of hepatocytes, such as APOA1 and APOC2.

Kill curves.

Cell line specific kill curves were used to identify the appropriate antibiotic concentrations by seeding cells at 50% confluence in 6 well plates and growing in various concentrations of antibiotics. The lowest concentration that killed all cells was chosen for subsequent use.

Protocol for Assay looking for viral entry/early replication factors.

An overview of the assay is shown in Supplemental Figure 1.

1. Day 0. Seed Huh7 or HepG2 cells at a density of $3x10^5$ cells into wells of a 6 well plate.
2. Day 0. Seed Huh7 or HepG2 cells at a density of $3x10^5$ cells into the insert (BD biosciences, San Jose, California). Grow the cells on the inserts in a 6 well plate with no cells growing on the bottom of the well.
3. Day 1. The next day (approximately 12 hours later), transfect 4 μg of the library DNA (lipofectamine) into the 6 well plates.
4. At the same time, transfect 1.6 ug transgenic HBV into the inserts (unless using a permanent transgenic HBV cell line).
5. Day 1. Six hours after transfection, wash the cells to remove the transfected material in both the 6 well plate and in the inserts. Wash well.
6. Day 1. Place the inserts into the 6 well plates.
7. Day 4. Remove the insert. Add geneticin (concentration for HepG2: 125 μg/mL ; Huh7: 75 μg/mL).
8. Day 5. Split each 6 well into a separate T25 flask. They need to be split to approximately 25% or less cellularity or the antibiotic selection wont work well. The cells will initially undergo a significant cell die off (varies, but typically 40-80%) in the presence of the antibiotic, but should recover and grow to confluence in the T25 flask. (geneticin concentration for HepG2: 250 μg/mL ; Huh7: 150 μg/mL).

   Note 1: The antibiotic added in step 7 is half concentration. The concentration is left at half concentration for 24 hours, and then is increased to full concentration after the split.

   Note 2: All subsequent splits are made based on the cell cellularity and are not based strictly on experimental days. Approximate days between the subsequent splits for

typical experiments are shown in Supplemental Figure 1, but there can be significant variation.

Note 2. Change the media and add fresh antibiotic every two to 3 days.

9. After 4 days in the full concentration of geneticin, add Blasticidin or Zeocin (concentration for HepG2: Zeocin_50 ug/mL, Blasticidin 0.25 ug/mL; Huh7: Zeocin 35 ug/mL, Blasticidin 0.25 ug/mL).

   Note 1: The transgenic antibiotic is added at half concentration at first.

10. The cells will slowly grow to confluence. When they are ready to split, add in full concentration of transgenic antibiotic about 24 hours before the split and then split into a T75 flask (each T25 goes to a T75). Also keep geneticin at full concentrations. Geneticin concentration for HepG2: 250ug/mL; for Huh7: 150ug/mL. Transgenic antibiotic concentrations for HepG2 are as follows: Zeocin 100ug/mL, Blasticidin 0.75ug/mL. Transgenic antibiotic concentrations for Huh7 are as follows: Zeocin 50ug/mL, Blasticidin 0.5ug/mL).

    Note 1: The cells will again undergo a significant cell die off (typically >90% of cells) after the split to a T75. The Huh7 cells are very fragile at this stage (when there are very few cells remaining) and sometimes benefit from splitting back into a very small sized container such as a 6 well plate. Sometimes they just don't make it. The HepG2 cells seem to be sturdier at very low cellularity and will slowly recover and grow to confluence.

    Note 2. Zeocin selection tends to take longer.

11. Once the cells form clones (can take several weeks), split them again back into a single T25. They will typically have some noticeable cell death at this step too (20-30%).
12. Once the cells are confluent, split them again.
    a. Take half for DNA extraction. The DNA can be extracted both by routine methods as well as with a miniprep protocol.
    b. Perform HBsAg ELISA on the supernatant extract to make sure it is HBV positive.
    c. Analyze the extracted DNA by real time PCR for HBV DNA levels.
    d. Use destination primers and perform PCR using DNA from step 12.a . Gel purify and sequence the DNA amplicon.

       Note 1. There should be a single or a few bright bands. If there is a smear, the cells did not select out or the PCR conditions need to be optimized.

e. Create real time primers that target the DNA identified in step 12.c.

General notes:

1. The antibiotic concentrations should be confirmed in your lab conditions with a kill curver before starting the experiments
2. The media is DMEM with high glucose and 10% FCS for all steps.
3. Transfection is with lipofectamine 2000 (Invitrogen).
4. After step 8, all of the steps are performed based on the flask/well cellularity and not on a strict date.
5. Antibiotics should not be old. If there is any uncertainty—ie approaching expiration date, get new ones. If the reconstitution date is more than a month or two old, make up new stock.
6. Analysis from DNA extracts from the early splits will show a smear on Dest PCR as the cells are not fully selected. If the PCR shows a smear in step 12, then the cells were not fully selected.
7. The supernatants from permanent cell lines used to seed the inserts contain approximately $5x10^4$ to $5x10^5$ copies of HBV DNA per ml, as determined by real time PCR. The 6 well plates are seeded with approximately $3x10^5$ cells for the assay, giving a "MOI" of approximately 0.5 to 1.3. This MOI is probably on the low side for optimal performance of the assay, but the assay works satisfactorily at this MOI.

Section 2, Table 1. Primers.

**Primer Name**	**Sequence, 5' to 3'**	**Amplicon size**	**Notes**
Destination F	CCACGCTGTTTTGACCTCCATAGAA	NA	Amplicon size will vary depending on size of cloned DNA. A smear indicates the cells are not fully selected.
Destination R	TAGCAGTTTTCCCAGTCACGACGTT		
Ampicillin F	TTT TGC CTT CCT GTT TTT GC	189	Used to quantitate library vector levels in supernatant. Can amplify genomic DNA so not useful if genomic DNA is present/abundant.
Ampicillin R	ATAATACCGCGCCACATAGC		
DDR2 DNA copy number 1F	GGAAGGGAGCAACAAATGAA	139	Used to quantitate genomic DNA levels.
DDR2 DNA copy number 1R	ACCTTCAGGCTGAGCTACCA		
HBV Is2 F	CATGCGTGGAACCTTTGTG	151	Used to quantitate HBV DNA levels. Targets 1233 to 1384 of HBV genome.
HBV Is2 R	TAGCAGCCATGGAAATGATG		
HBV For4	CCTATGGGAGTGGGCCTCA	122	Used to quantitate HBV DNA levels. Targets 637 to 759 of HBV genome.
HBV Rev7	CCCCAATACCACATCATCCATATA		
pcDNA 3.1 1F	TAGTTGCCAGCCATCTGTTG	104	Use to quantitate amount of vector. This is the vector that is used to clone the 1.3X transgenic virus
pcDNA 3.1 1R	GCGATGCAATTTCCTCATTT		

REFERENCES

1. Chen A, Kao YF, Brown CM. Translation of the first upstream ORF in the hepatitis B virus pregenomic RNA modulates translation at the core and polymerase initiation codons. Nucleic Acids Res 2005; 33:1169-81.

2. Vivekanandan P, Daniel HD, Kannangai R, Martinez-Murillo F, Torbenson M. Hepatitis B virus replication induces methylation of both host and viral DNA. J Virol 2010; 84:4321-9.

3. Vivekanandan P, Thomas D, Torbenson M. Hepatitis B viral DNA is methylated in liver tissues. J Viral Hepat 2008; 15:103-7.

4. Vivekanandan P, Thomas D, Torbenson M. Methylation regulates hepatitis B viral protein expression. J Infect Dis 2009; 199:1286-91.

5. Gunther S, Li BC, Miska S*, et al.* A novel method for efficient amplification of whole hepatitis B virus genomes permits rapid functional analysis and reveals deletion mutants in immunosuppressed patients. J Virol 1995; 69:5437-44.

6. Siebenkotten G, Leyendeckers H, Christine R, Radbruch A. Isolation of plasmid DNA from mammalian cells with QIAprep. Qiagen News and Product Application 1995; 2:11-3.

7. Zanella I, Rossini A, Domenighini D, Albertini A, Cariani E. Quantitative analysis of hepatitis B virus DNA by real-lime amplification. Eur J Clin Microbiol Infect Dis 2002; 21:22-6.
